# Supplementary material for: A cryo-electron microscopy support film formed by 2D crystals of hydrophobin HFBI
Source: Nat Commun. 2021 Dec 14;12:7257. doi: 10.1038/s41467-021-27596-8 (PMC8671466; doi:10.1038/s41467-021-27596-8)
Supplement: Supplementary file 1 — Supplementary Information [file 41467_2021_27596_MOESM1_ESM.pdf]

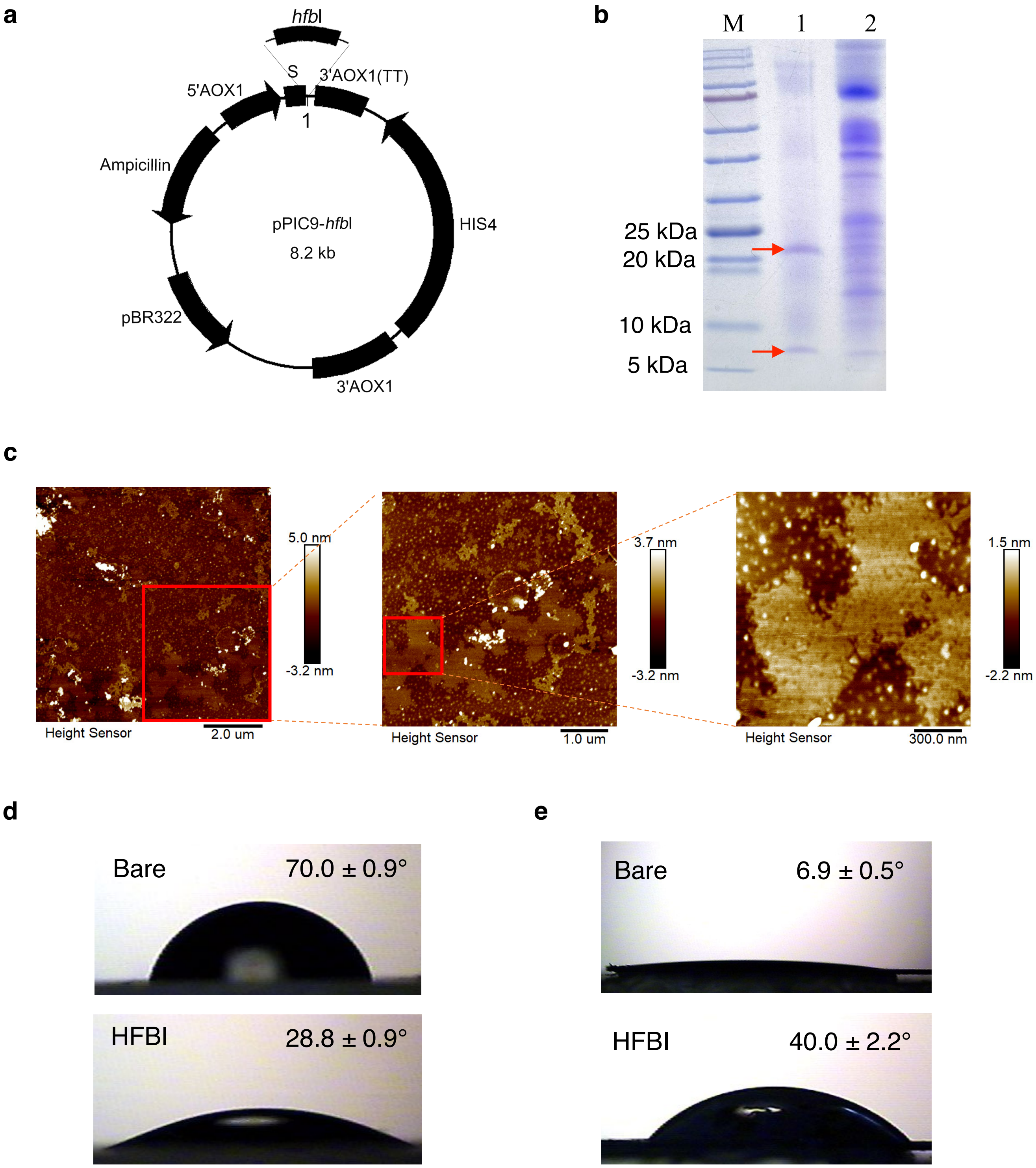
a
hfbI
S
3'AOX1(TT)
5'AOX1
1
Ampicillin
pPIC9-hfbI
8.2 kb
HIS4
pBR322
3'AOX1
b
M
1
2
25 kDa
20 kDa
10 kDa
5 kDa
c
5.0 nm
-3.2 nm
Height Sensor
2.0 um
3.7 nm
-3.2 nm
Height Sensor
1.0 um
1.5 nm
-2.2 nm
Height Sensor
300.0 nm
d
Bare
70.0 ± 0.9°
HFBI
28.8 ± 0.9°
e
Bare
6.9 ± 0.5°
HFBI
40.0 ± 2.2°

f

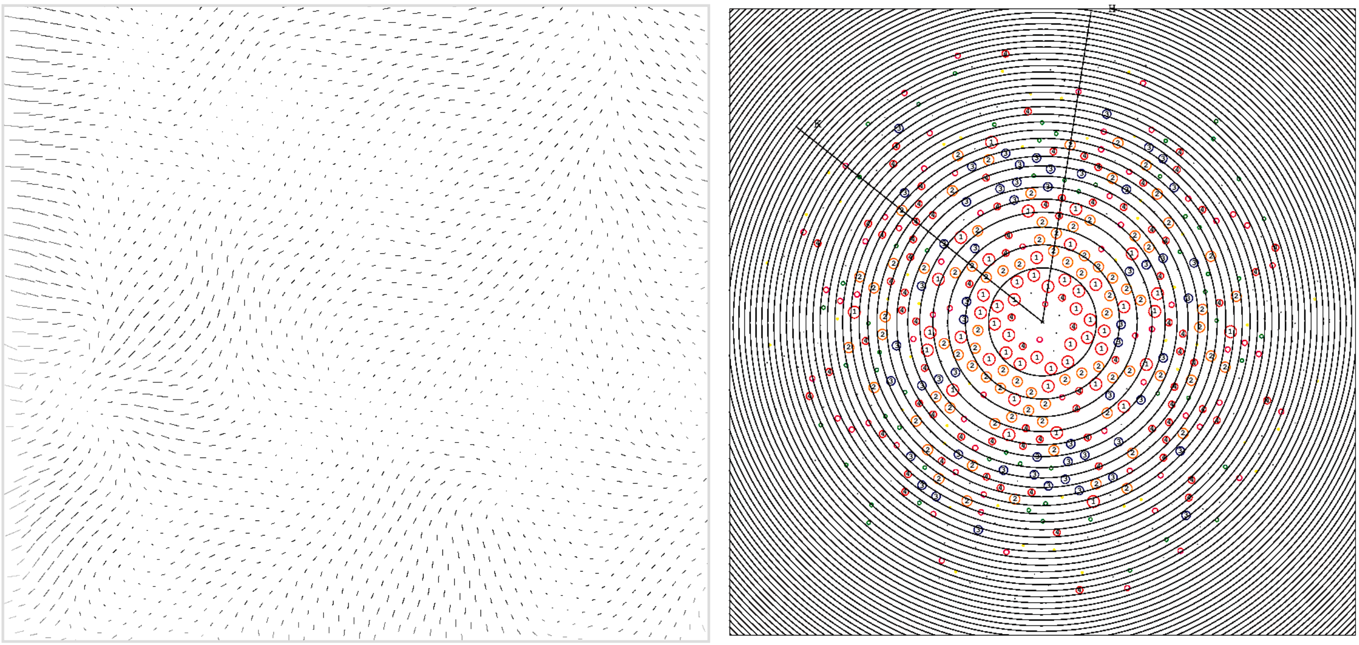

Supplementary Fig. 1 | Preparation and characterization of HFBI film. a, Schematic map of the plasmid pPIC9-hfbI used for recombinant expression of hydrophobin HFBI. **b**, SDS PAGE of purified HFBI. Lane 1, purified HFBI that comprises monomer and trimer indicated by the red arrows, respectively. Lane 2, raw HFBI extracts. M, molecular weight marker. This has been repeated independently more than three times and all yielded similar results. **c**, Atomic force microscopic images of HFBI film coated mica in different magnifications. Images are coloured according to the parameters of measured heights that were calibrated at the region of uncoated mica. See more experimental details in Methods. This experiment was repeated independently more than three times with similar results. **d**, Water contact angle measurements of the surfaces of bare (up) and HFBI film (bottom) coated siliconized glass. **e**, Water contact angle measurements of the surfaces of bare (up) and HFBI film (bottom) coated mica. See more experimental details in Methods. **f**, The cryo-EM micrograph of HFBI film was processed using 2dx package [1]. The crystal distortion vector (left) from unbending analysis and the IQ plot [2] (right) of diffraction spots after distortion correction are displayed here. In the IQ plot, each diffraction spot is shown as a circle with the radius indicating the signal to noise ratio for that spot. The diffraction spots are labeled with the corresponding IQ value.

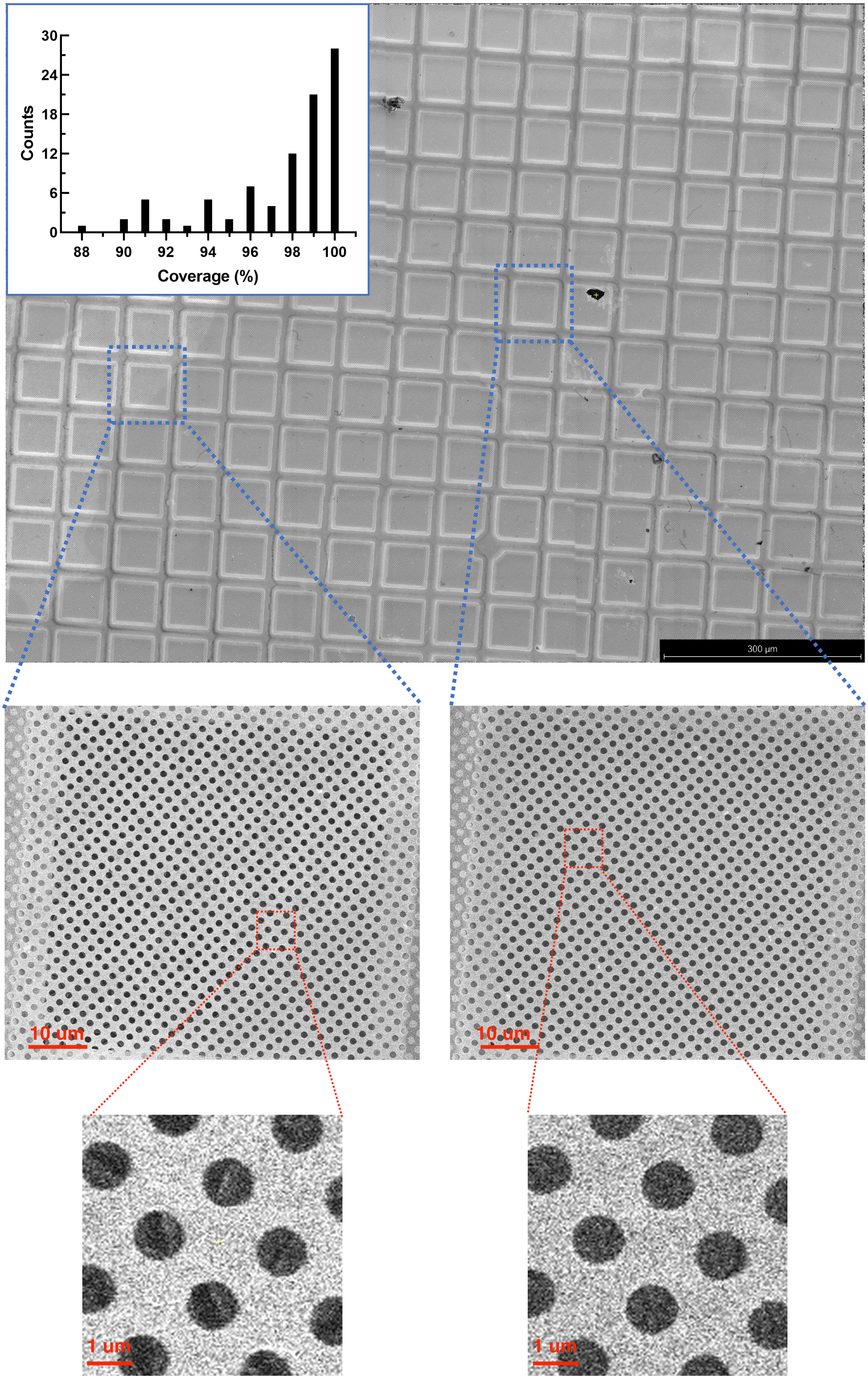

Counts
Coverage (%)
0
6
12
18
24
30
88
90
92
94
96
98
100
300 µm
10 um
10 um
1 um
1 um

Supplementary Fig. 2 | Coverage of HFBI film on the grid examined by scanning electron microscopy (SEM). The dried HFBI film covered ANTA foil gird was examined by SEM at room temperature. SEM images were acquired using ThermoFisher Scientific Aquilos II that was operated at 2 keV accelerate volage and 13 pA current with an ETD detector in the secondary electron mode and the imaging parameters of 1 μs dwell time and 9.0 mm working distance. The montage SEM image of the whole grid was acquired and shown on the top with two regions zoomed in on the bottom, one region (left) representing the broken HFBI film area and another (right) for the integral HFBI film area. The coverage of integral HFBI film was counted from 90 squares of three batches of grids and is shown on the inset of the top. It is clear the averaged coverage of HFBI film is higher than 90%.

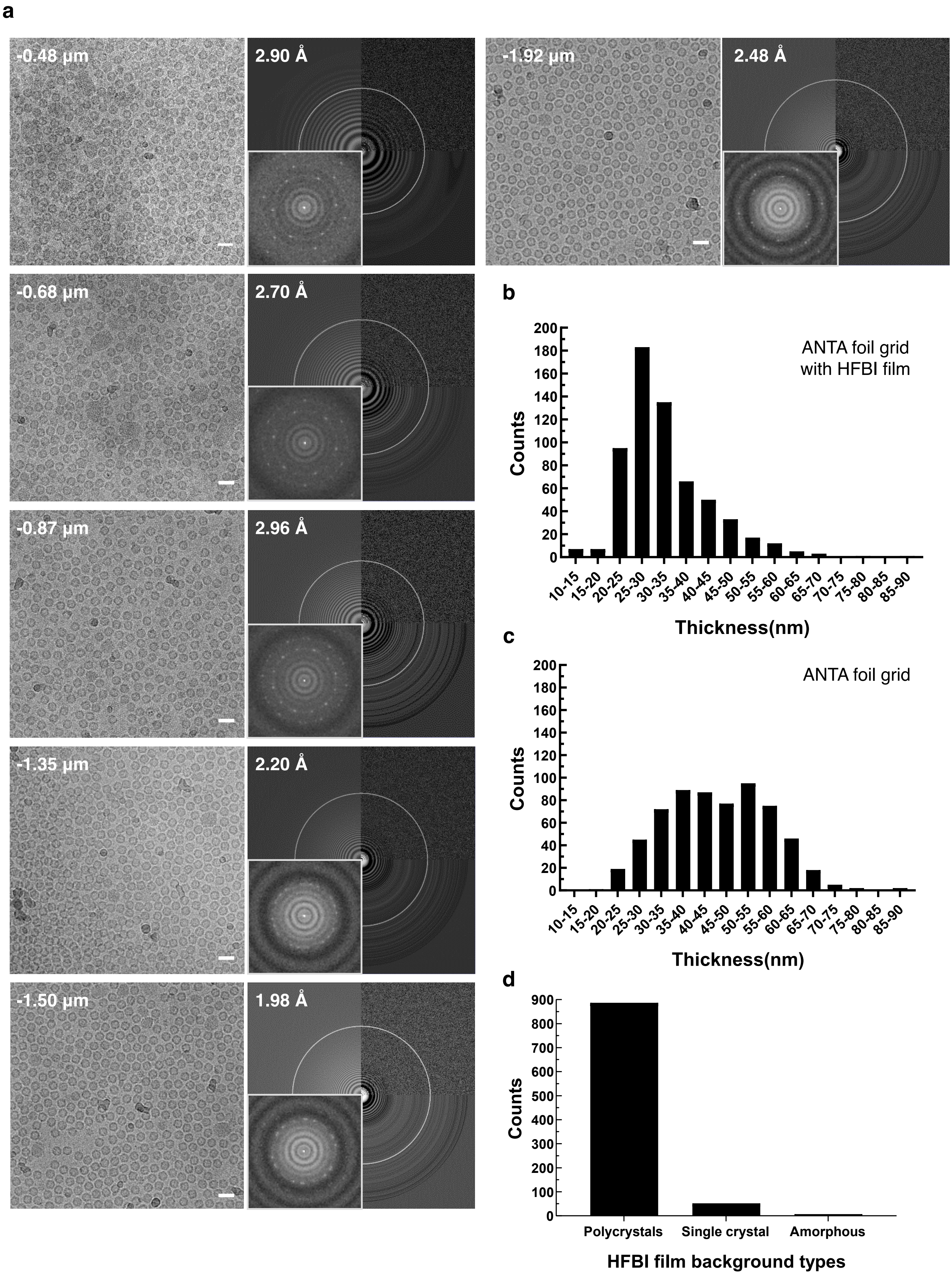
a
-0.48 μm
2.90 Å
-1.92 μm
2.48 Å
-0.68 μm
2.70 Å
-0.87 μm
2.96 Å
-1.35 μm
2.20 Å
-1.50 μm
1.98 Å
b
ANTA foil grid
with HFBI film
Counts
Thickness(nm)
0
20
40
60
80
100
120
140
160
180
200
10-15
15-20
20-25
25-30
30-35
35-40
40-45
45-50
50-55
55-60
60-65
65-70
70-75
75-80
80-85
85-90
c
ANTA foil grid
Counts
Thickness(nm)
d
Counts
0
100
200
300
400
500
600
700
800
900
Polycrystals
Single crystal
Amorphous
HFBI film background types

Supplementary Fig. 3 | Cryo-EM experiments of human apoferritin cryo-vitrified using HFBI film covered ANTA grid. a, Serials of defocused (-0.48 *μ*m ~ -1.92 *μ*m) cryo-EM micrographs (Scale bar, 20 nm) with the corresponding Gctf [3] computed fitted power spectrums shown aside. The defocus value of each micrograph and the Gctf estimated reachable resolution in each power spectrum are labeled accordingly. Each zoomed in power spectrum in the central region to show diffraction spots of HFBI film support is shown as an inset accordingly. **b,** Statistics of ice thickness from cryo-EM dataset (> 800 micrographs) of human apoferritin that was cryo-vitrified using HFBI film covered ANTA foil grid. **c,** Statistics of ice thickness from cryo-EM dataset (> 600 micrographs) of human apoferritin that was cryo-vitrified using ANTA foil grid. Both cryo-EM datasets (**b** and **c**) were collected using the energy filter (Gatan BioQuantum) with a slit width of 20 eV. One electron micrograph at the empty region was recorded at the beginning of each data collection. Then the ice thickness (t_1) of each micrograph was calculated using the formula $t_1 = \Lambda \ln(I_0/I_1)$, where I_1 represents the mean intensity of the target micrograph, I_0 represents the mean intensity of the micrograph taken at the empty region, and Λ represents the inelastic mean free path of 200 keV electron [4]. **d,** Statistics of types (polycrystal, single crystal and amorphous) of HFBI film from cryo-EM dataset (> 900 micrographs) of human apoferritin that was cryo-vitrified using HFBI film covered ANTA foil grid. The type of HFBI film was determined by examining the diffraction spots in the central region of the corresponding power spectrum.

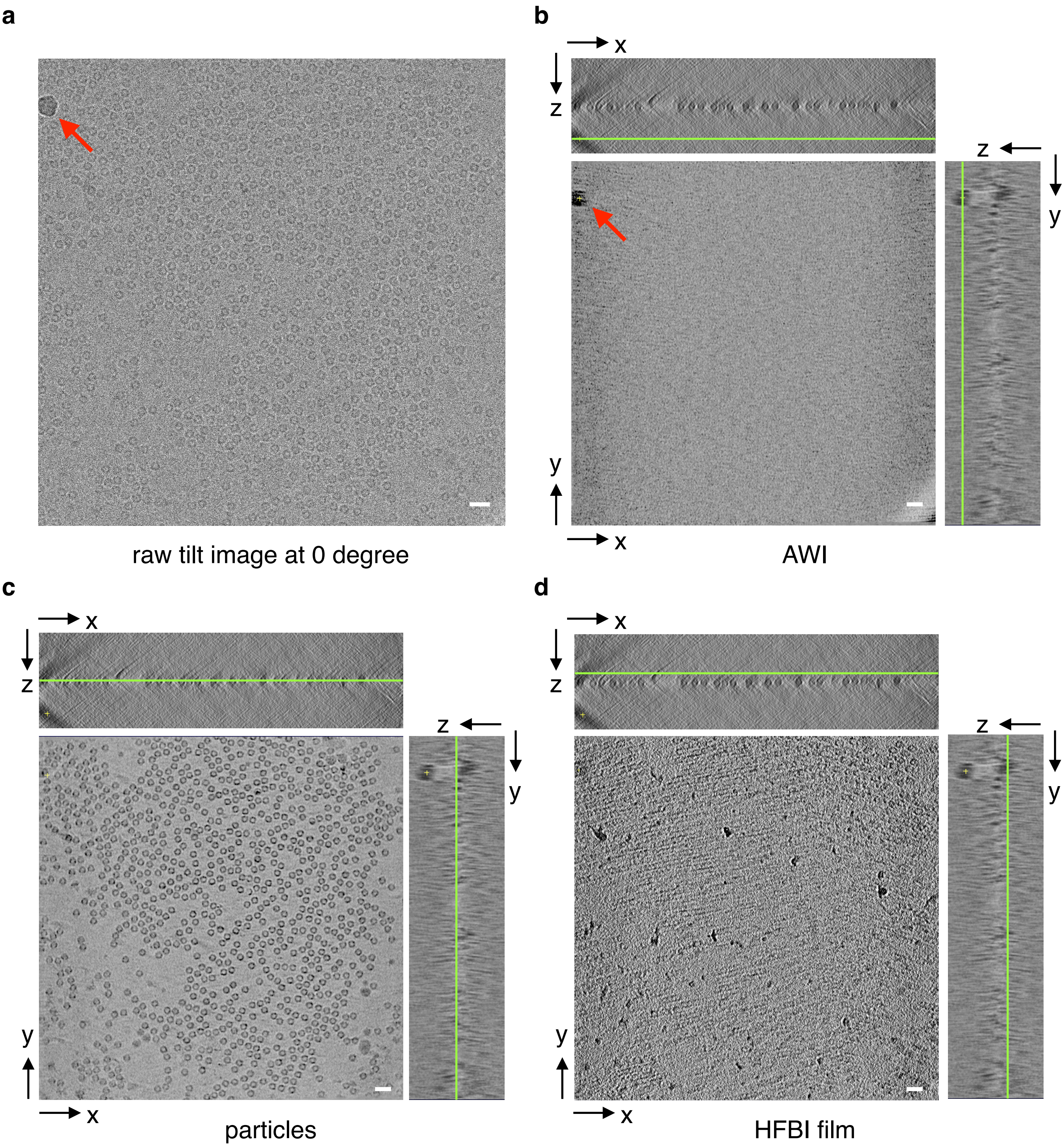

Supplementary Fig. 4 | Cryo electron tomographic reconstruction of human apoferritin cryo-vitrified using HFBI film covered ANTA foil grid. a, The original electron micrograph at the zero degree of tilt angle. One ice contamination is indicated by the red arrow. **b**, **c**, **d**, The XY, XZ and YZ slices of tomogram at the positions of air-water interface (AWI, **b**), the layer of apoferritin particles (**c**) and the layer of HFBI film (**d**). The location of AWI is indicated by the existence of the ice contamination (the red arrow in **b**). The location of HFBI film is indicated by the existence of contaminations on the back side of the film (**d**). It is clear almost all the apoferritin particles are adhering to the HFBI film and away from AWI. The yellow crosses indicate the position of ice contamination at AWI and were generated in 3dmod program of IMOD [5]. The green lines represent positions of XY slices located at the sections of YZ and ZX planes of the tomogram. Scale bar, 40 nm.

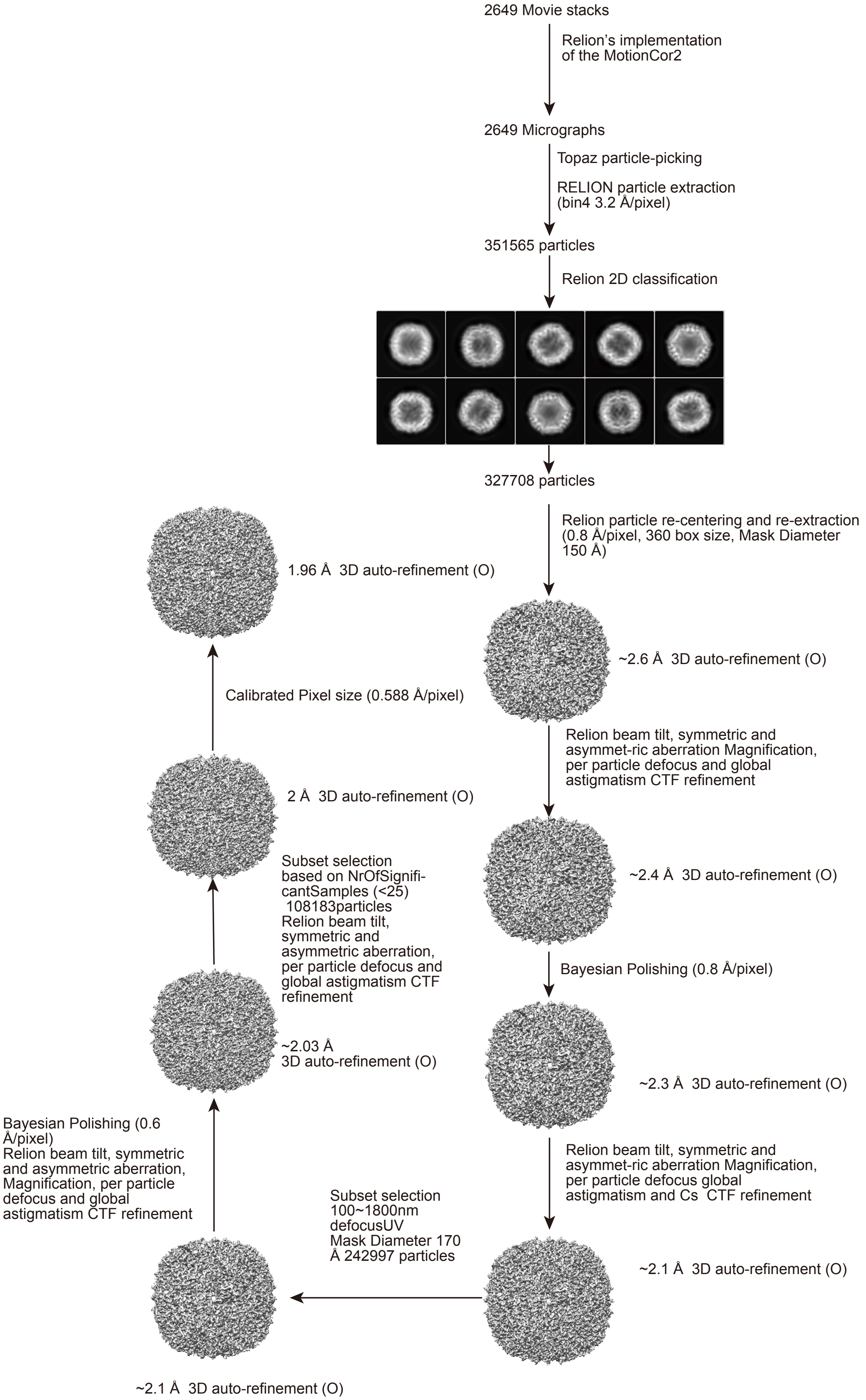

Supplementary Fig. 5 I Cryo-EM image processing workflow of human apoferritin cryo-vitrified using HFBI film covered ANTA foil grid.

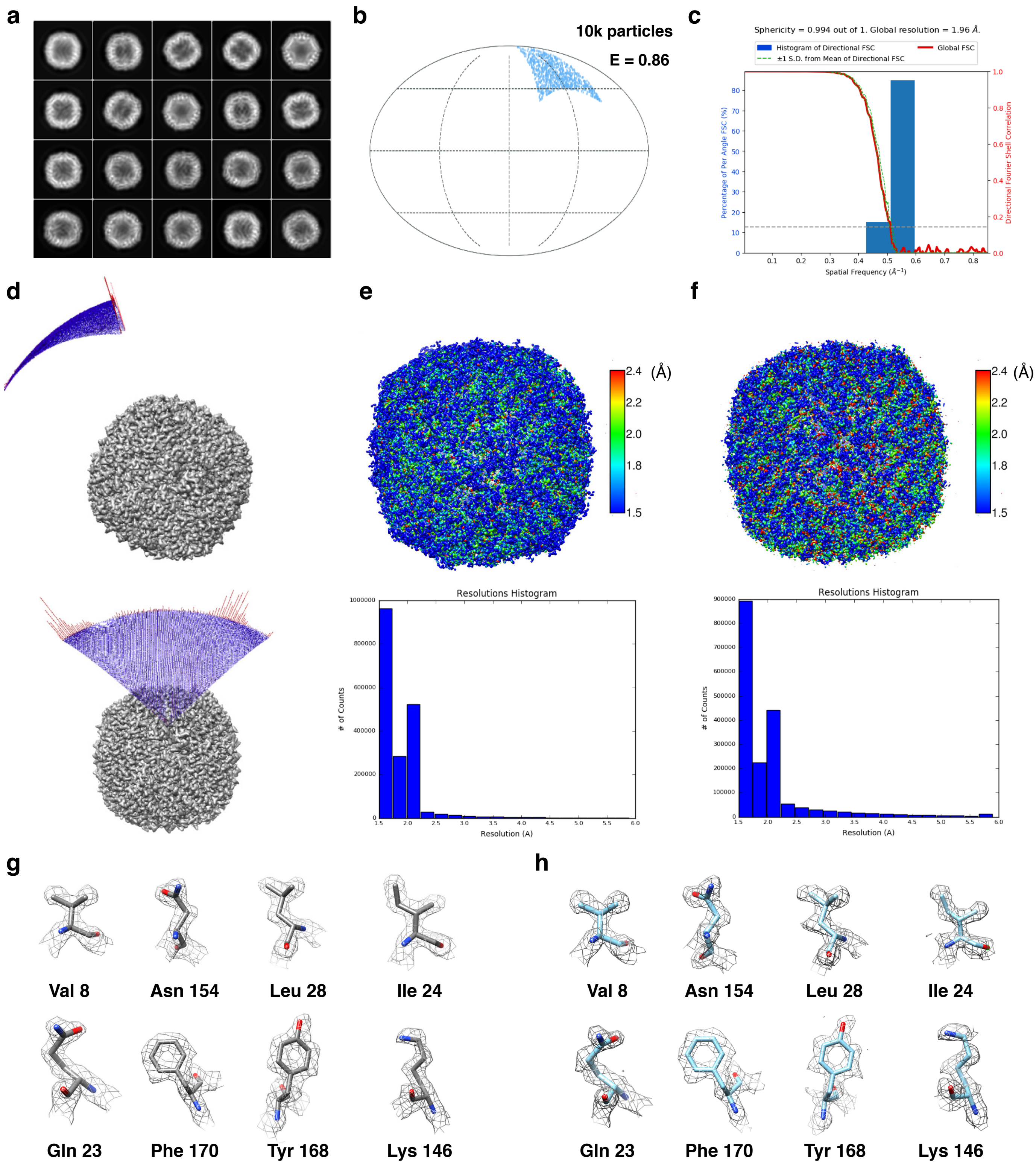

Supplementary Fig. 6 I Cryo-EM structure determination of human apoferritin cryo-vitrified using HFBI film covered ANTA foil grid. a, The representative of 2D class averages. **b**, The orientation distribution of apoferritin particles with the cryo-EF value of 0.86 calculated using 10,000 particles. **c,** 3D-FSC plot of cryo-EM reconstruction. **d,** Plots showing the Euler angle distribution of the particles. **e**, Cryo-EM map of apoferritin (this work), which was generated after density modification[6] using PHENIX (v1.19-4092)[7], is coloured according to the local resolution (from 2.4 Å in red to 1.5 Å in blue) computed by DeepRes[8]. **f**, Cryo-EM map of apoferritin (EMDB entry EMD-21024)[9] is coloured according to the local resolution (from 2.4 Å in red to 1.5 Å in blue) using DeepRes. **g, h,** Representative densities of selected residues from cryo-EM map of apoferritin (this work) before (**g**) and after (**h**) density modification.

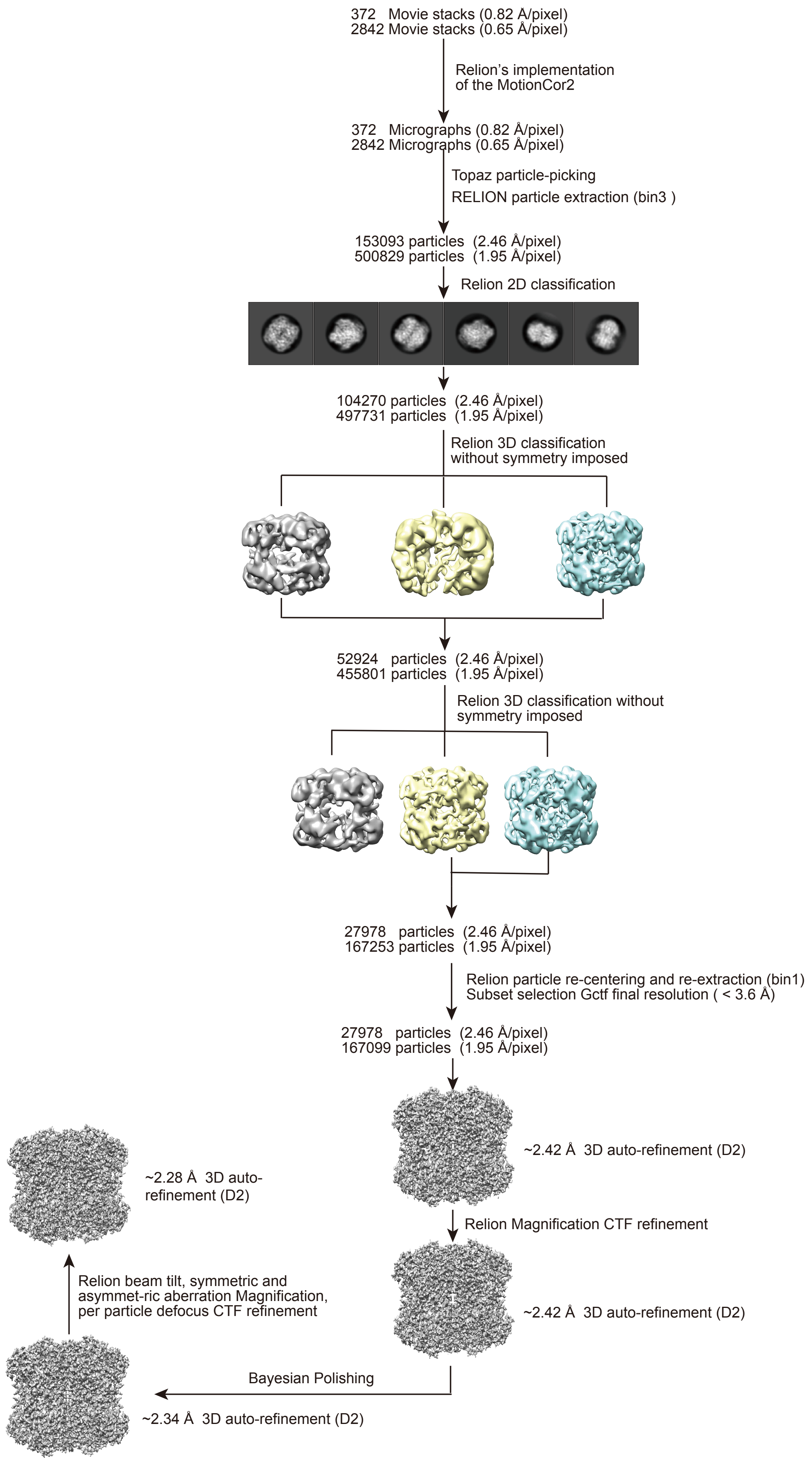

Supplementary Fig. 7 l Cryo-EM image processing workflow of catalase cryo-vitrified using HFBI film covered ANTA foil grid.

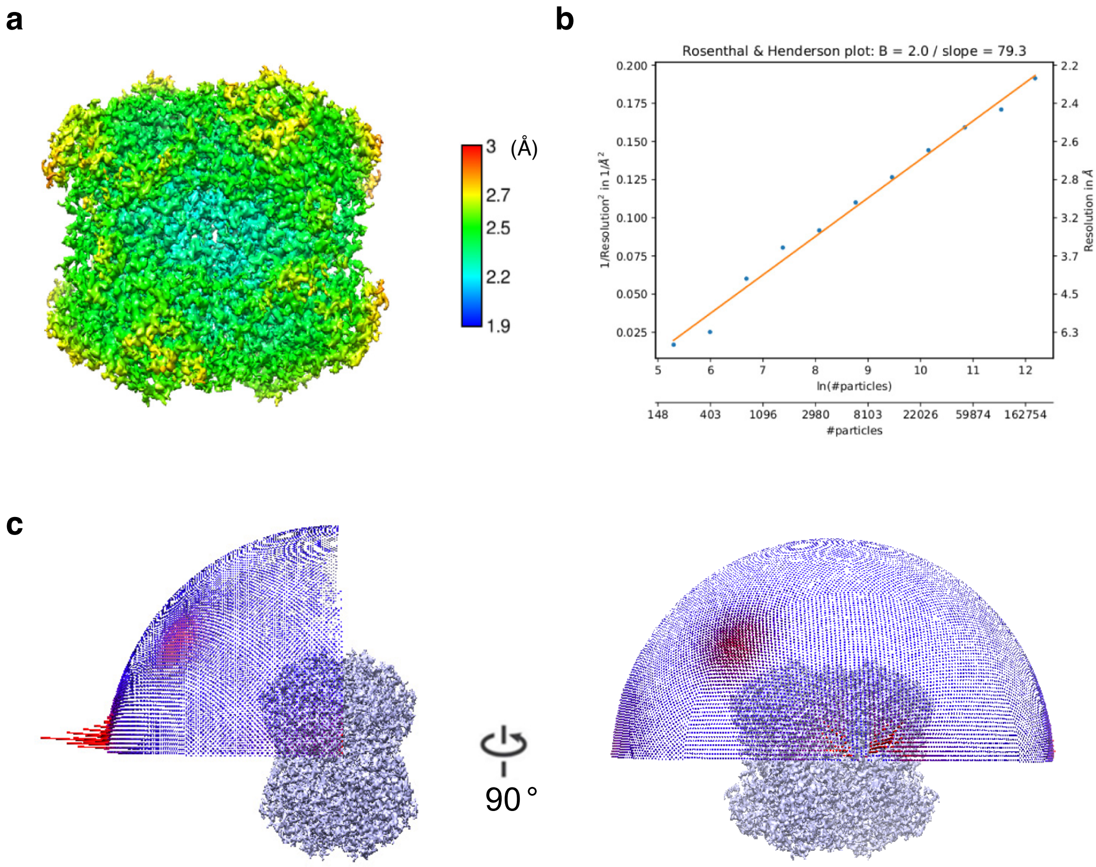

Supplementary Fig. 8 I Cryo-EM structure determination of catalase cryo-vitrified using HFBI film covered ANTA foil grid. a, Cryo-EM map of catalase is coloured according to the local resolution (from 3.0 Å in red to 1.9 Å in blue). **b,** Rosenthal-Henderson plot with the estimated B-factor of 79.3 Å. **c,** Plots showing the Euler angle distribution of the particles.

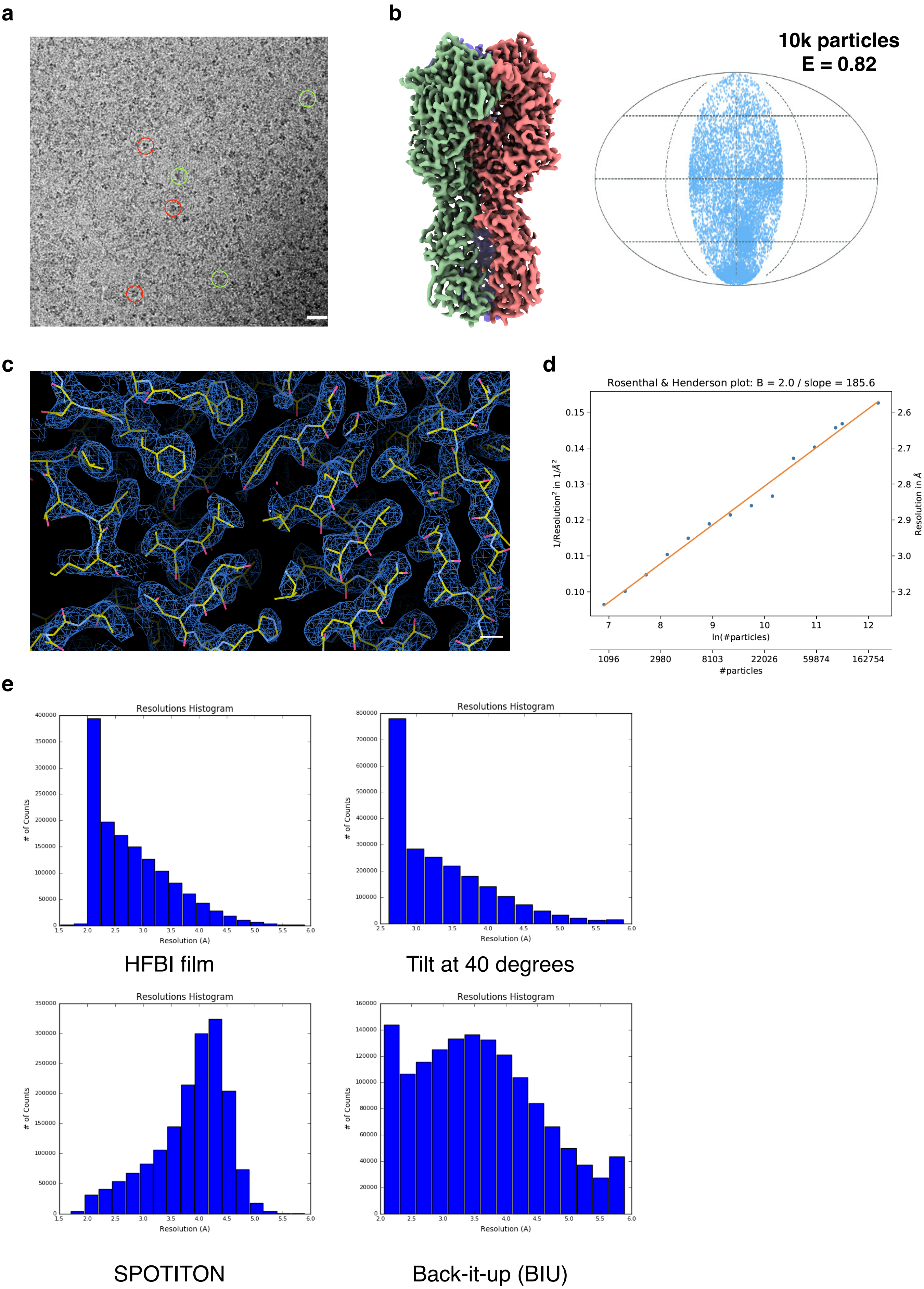
a
b
10k particles
E = 0.82
c
d
Rosenthal & Henderson plot: B = 2.0 / slope = 185.6
1/Resolution² in 1/Å²
Resolution in Å
ln(#particles)
#particles
1096
2980
8103
22026
59874
162754
e
Resolutions Histogram
# of Counts
Resolution (A)
HFBI film
Tilt at 40 degrees
SPOTITON
Back-it-up (BIU)

Supplementary Fig. 9 | Cryo-EM structure determination of influenza HA trimer cryo-vitrified using HFBI film covered ANTA foil grid. a, The representative cryo-EM micrograph of influenza HA trimer. Scale bar, 20 nm. The representative top views and tilt/side views are indicated by red and green circles, respectively. **b,** Cryo-EM map of influenza HA trimer at 2.56 Å resolution and its cryo-EF value (0.82) calculated using 10,000 particles. Three HA monomers in the cryo-EM map are coloured by green, pink and magenta, respectively. **c,** The representative region of cryo-EM map is zoomed in with the atomic model fitted. Scale bar, 2 Å. **d,** Rosenthal-Henderson plot with the estimated B-factor of 185.6 Å. **e,** DeepRes[8] computed local resolution histograms of cryo-EM maps of influenza HA trimer, which were determined by different approaches including HFBI film (this work), "Tilt at 40 degrees"[10, 11], SPOTITON[12] and Back-it-up (BIU)[13] approaches. See **Fig. 4** for more details.

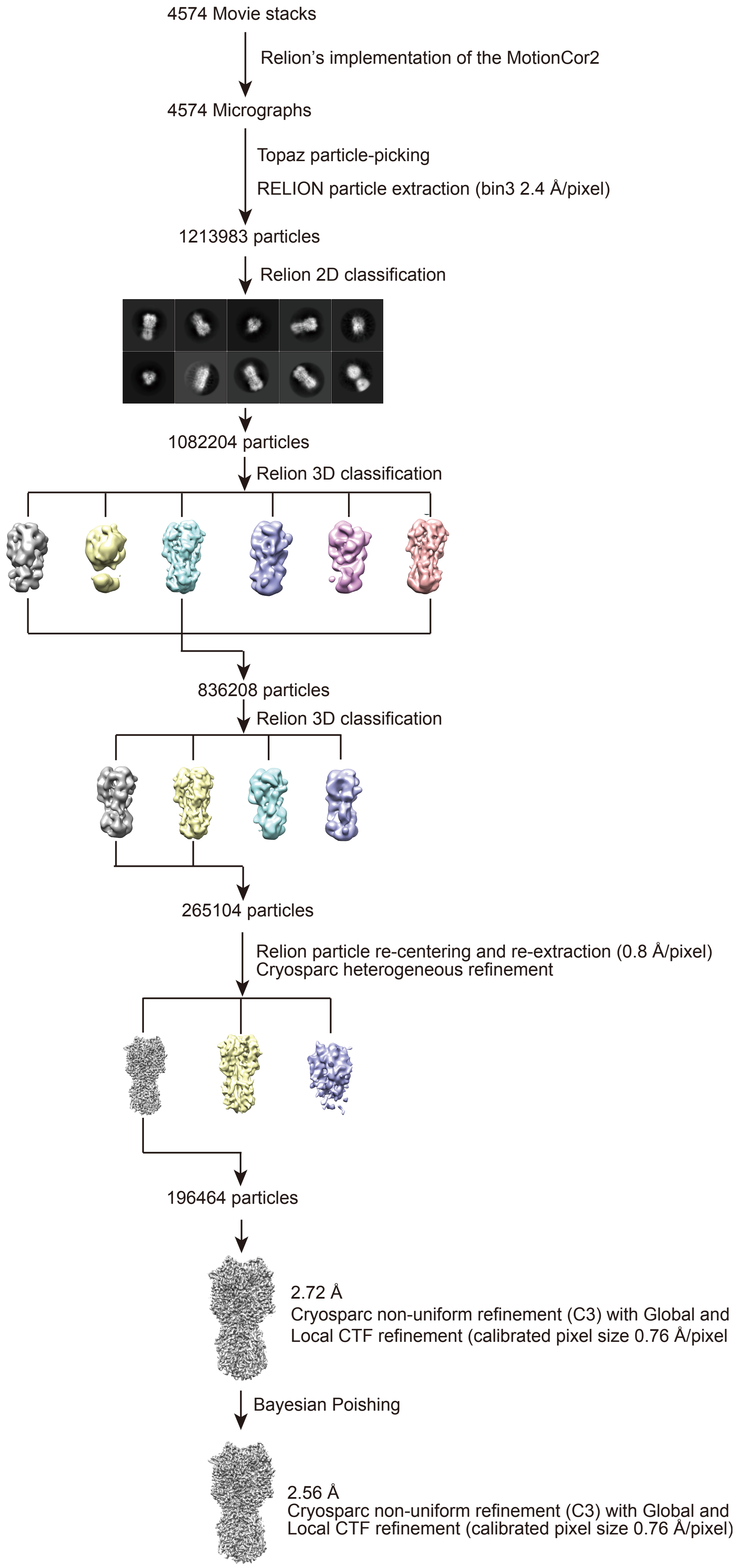

Supplementary Fig. 10 l Cryo-EM image processing workflow of influenza HA trimer cryo-vitrified using HFBI film covered ANTA foil grid.

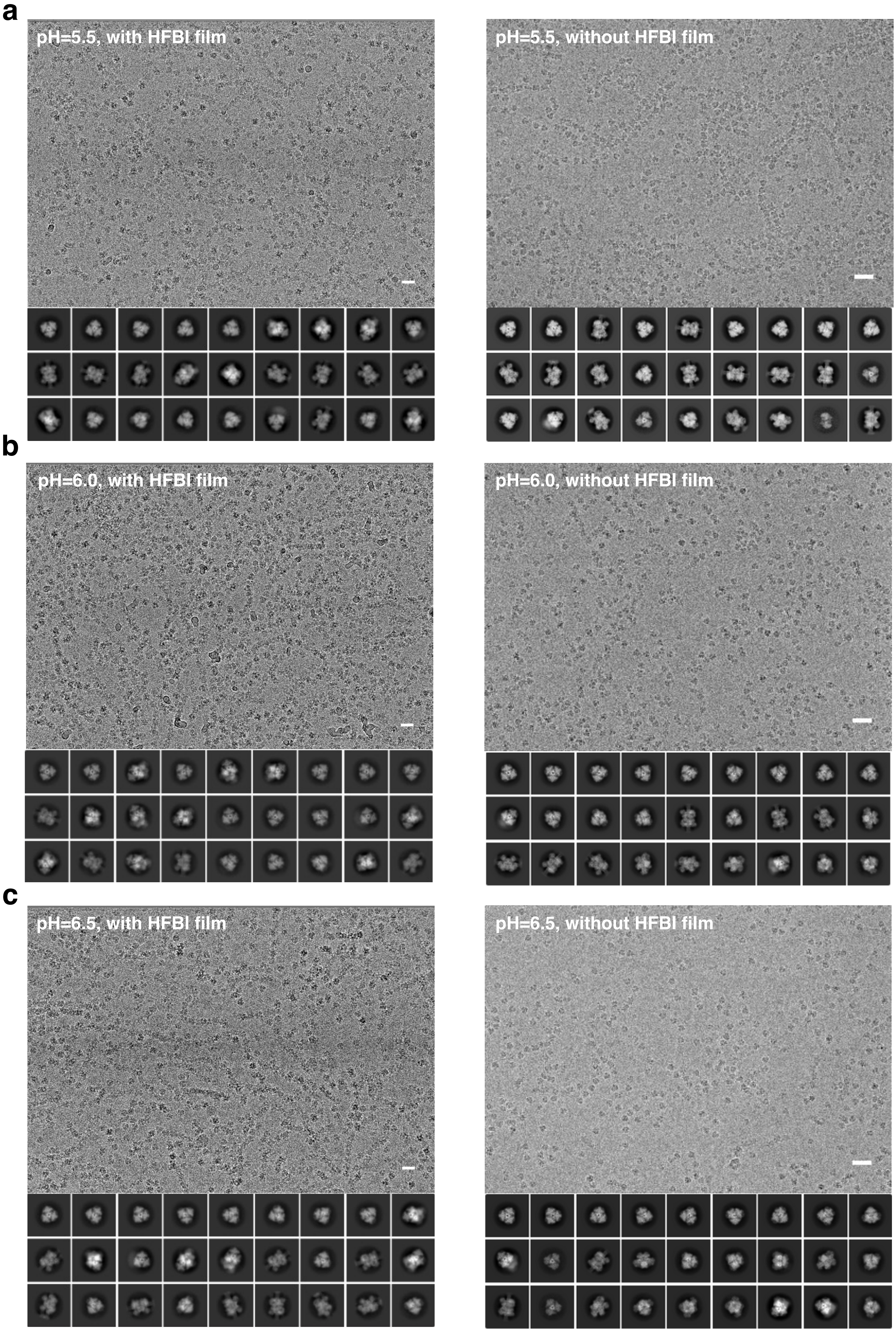
a
pH=5.5, with HFBI film
pH=5.5, without HFBI film
b
pH=6.0, with HFBI film
pH=6.0, without HFBI film
c
pH=6.5, with HFBI film
pH=6.5, without HFBI film

d

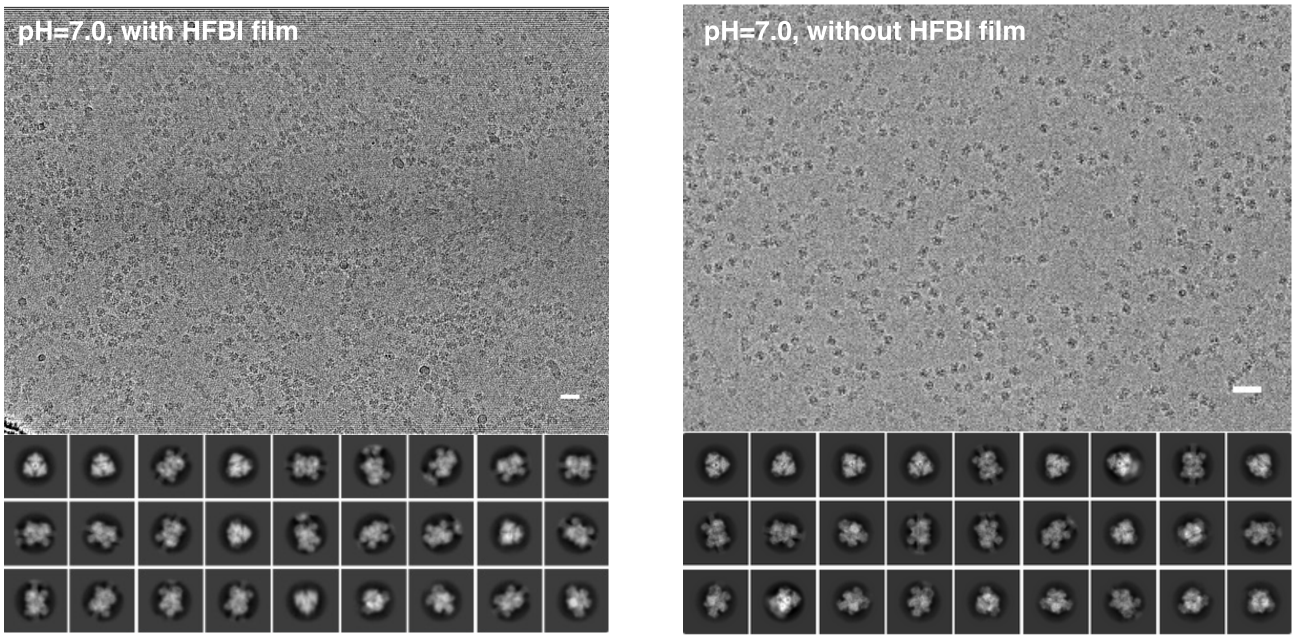
pH=7.0, with HFBI film
pH=7.0, without HFBI film

e

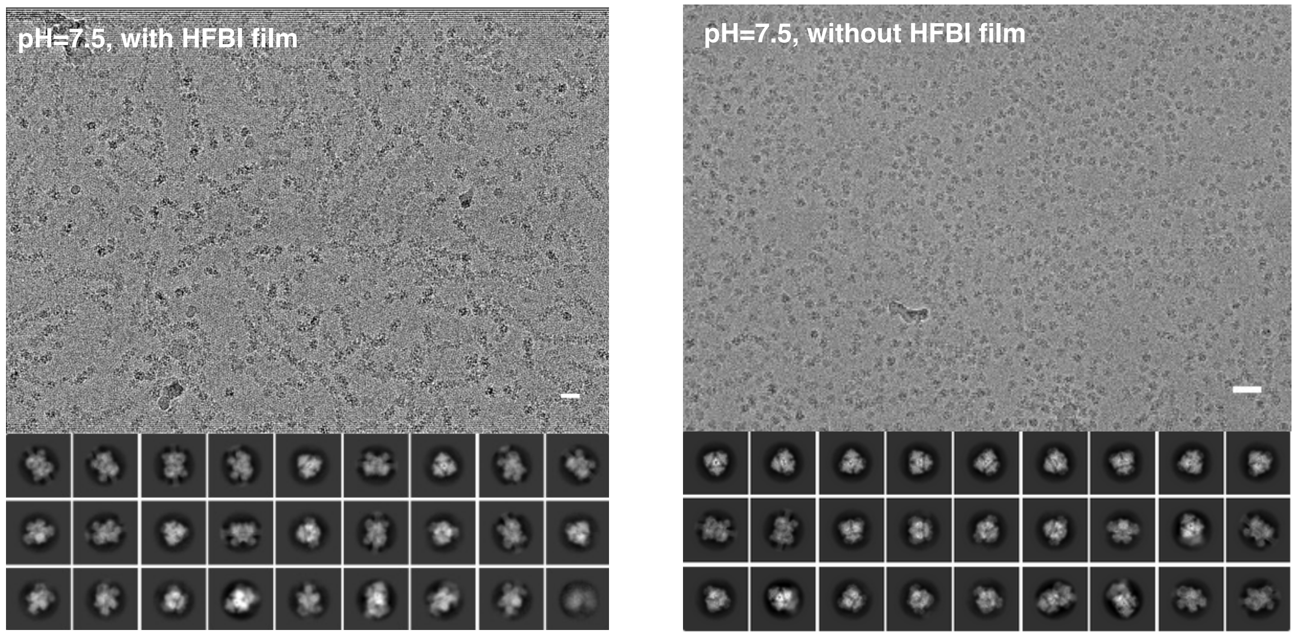
pH=7.5, with HFBI film
pH=7.5, without HFBI film

f

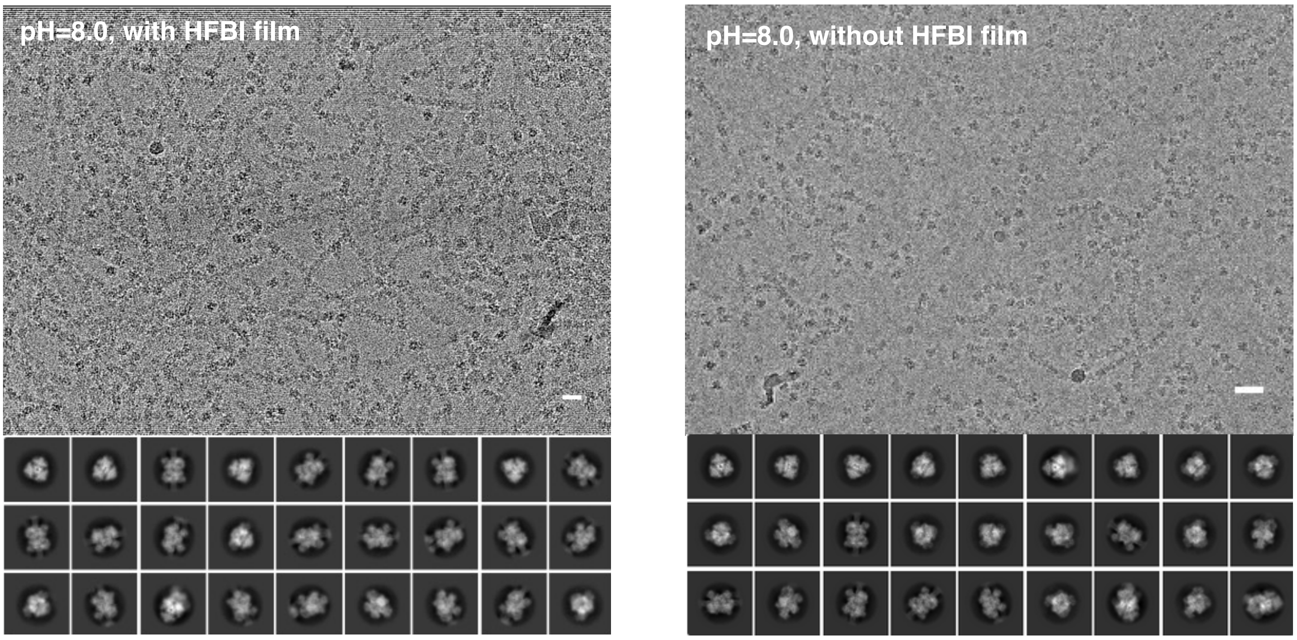
pH=8.0, with HFBI film
pH=8.0, without HFBI film

g

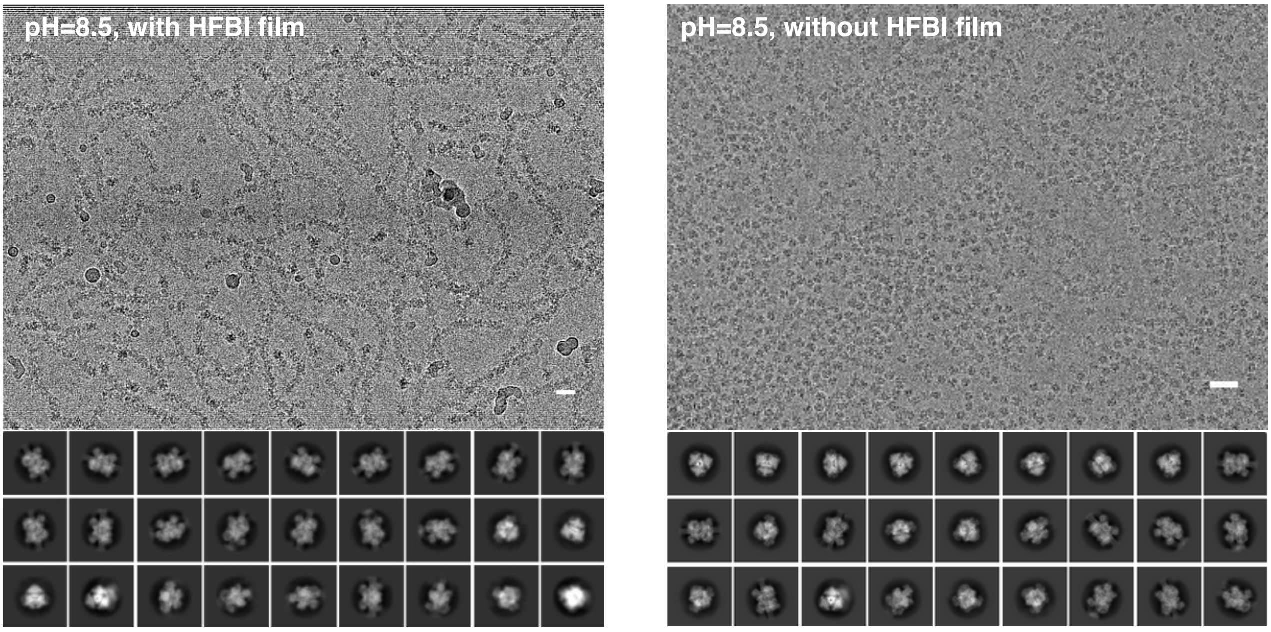

Supplementary Fig. 11 | Representative cryo-EM micrographs of GDH and corresponding 2D classifications in different buffer conditions. The GDH specimens were buffered in PBS (phosphate buffered saline) with pH 5.5 (**a**), 6.0 (**b**), 6.5 (**c**), 7.0 (**d**), 7.5 (**e**), and 8.0 (**f**), in Tris buffer (20mM Tris-HCl and 150 mM NaCl, pH 8.5, **g**). Each left panel shows the cryo-EM micrograph and the corresponding 2D classification of GDH cryo-vitrified using the HFBI film covered ANTA foil grids. Each right panel shows the cryo-EM micrograph and the corresponding 2D classification of GDH cryo-vitrified using holey ANTA foil grids. The class averages in each 2D classification are arranged with the descending order according to the percentage of number of particles in each class. Scale bar, 20 nm.

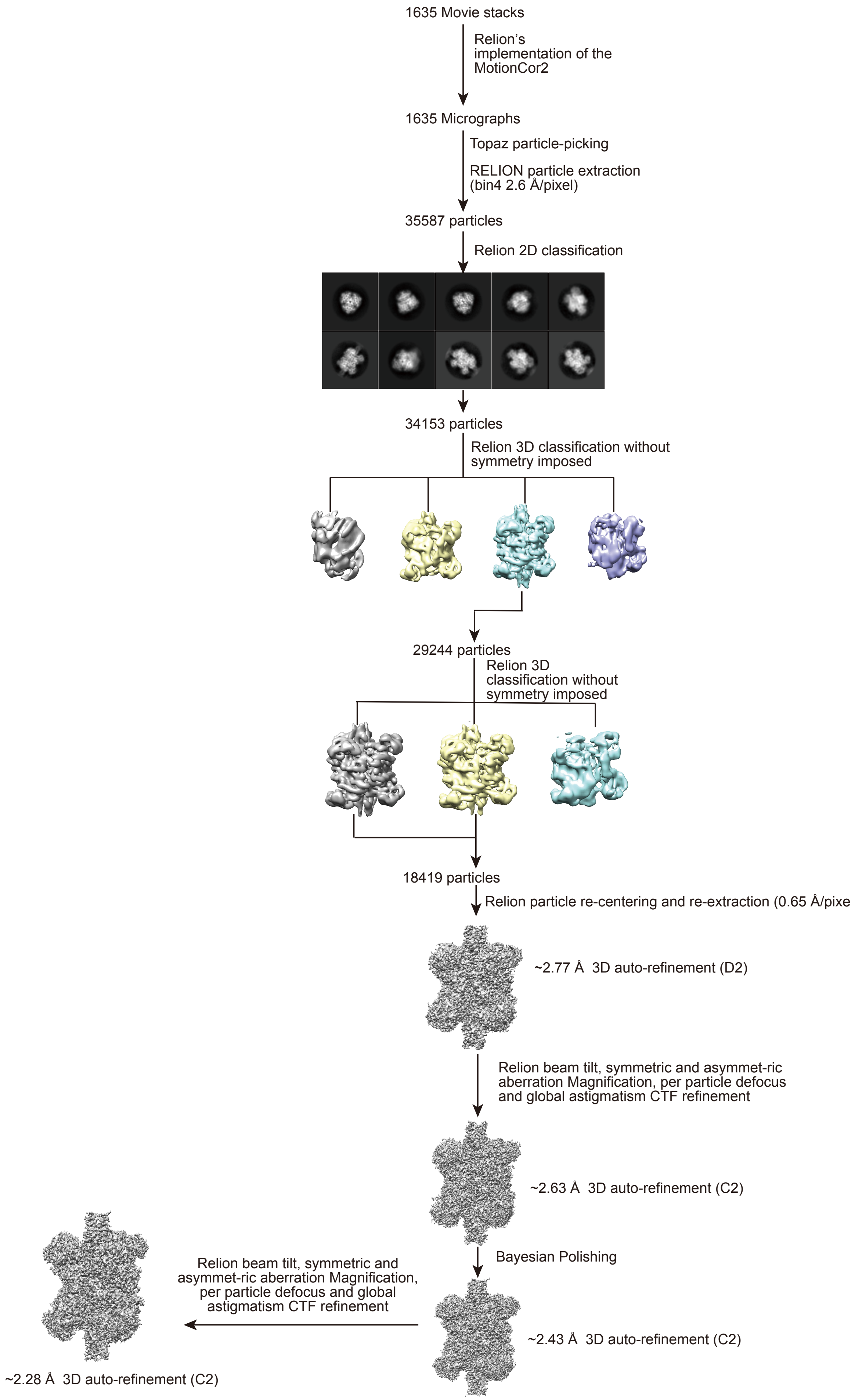

Supplementary Fig. 12 I Cryo-EM image processing workflow of GDH buffered in PBS (pH 7.5) and cryo-vitrified using HFBI film covered ANTA foil grid.

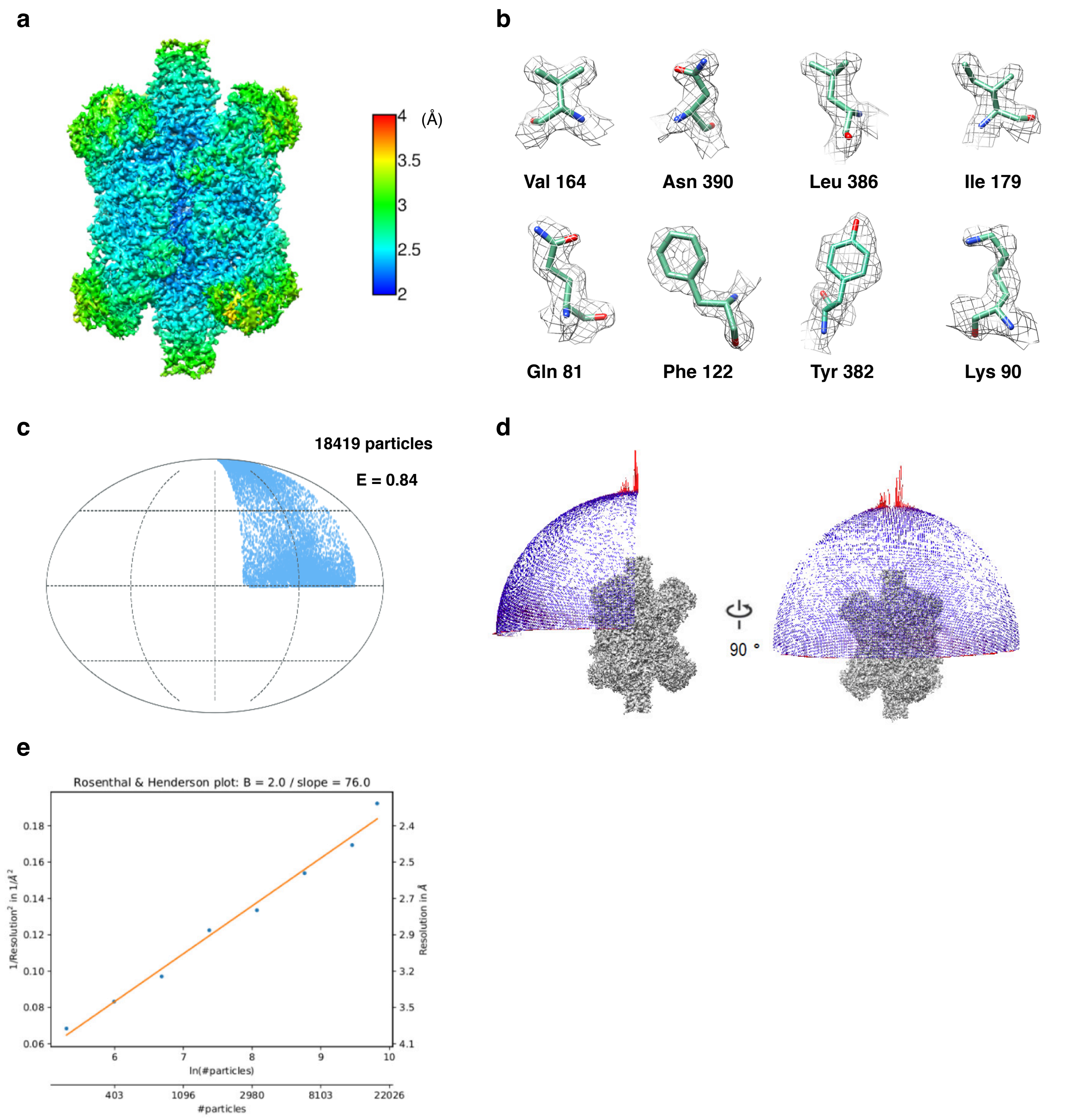

Supplementary Fig. 13 l Cryo-EM structure determination of pH 7.5 buffered GDH cryo-vitrified using HFBI film covered ANTA foil grid. a, Cryo-EM map of GDH is coloured according to the local resolution (from 4.0 Å in red to 2.0 Å in blue). **b,** Representative densities of selected residues from cryo-EM map of GDH. **c,** The orientation distribution of GDH particles with the cryo-EF value of 0.84 calculated using 18,419 particles. **d**, Plots showing the Euler angle distribution of the particles. **e**, Rosenthal-Henderson plot with the estimated B-factor of 76.0 Å.

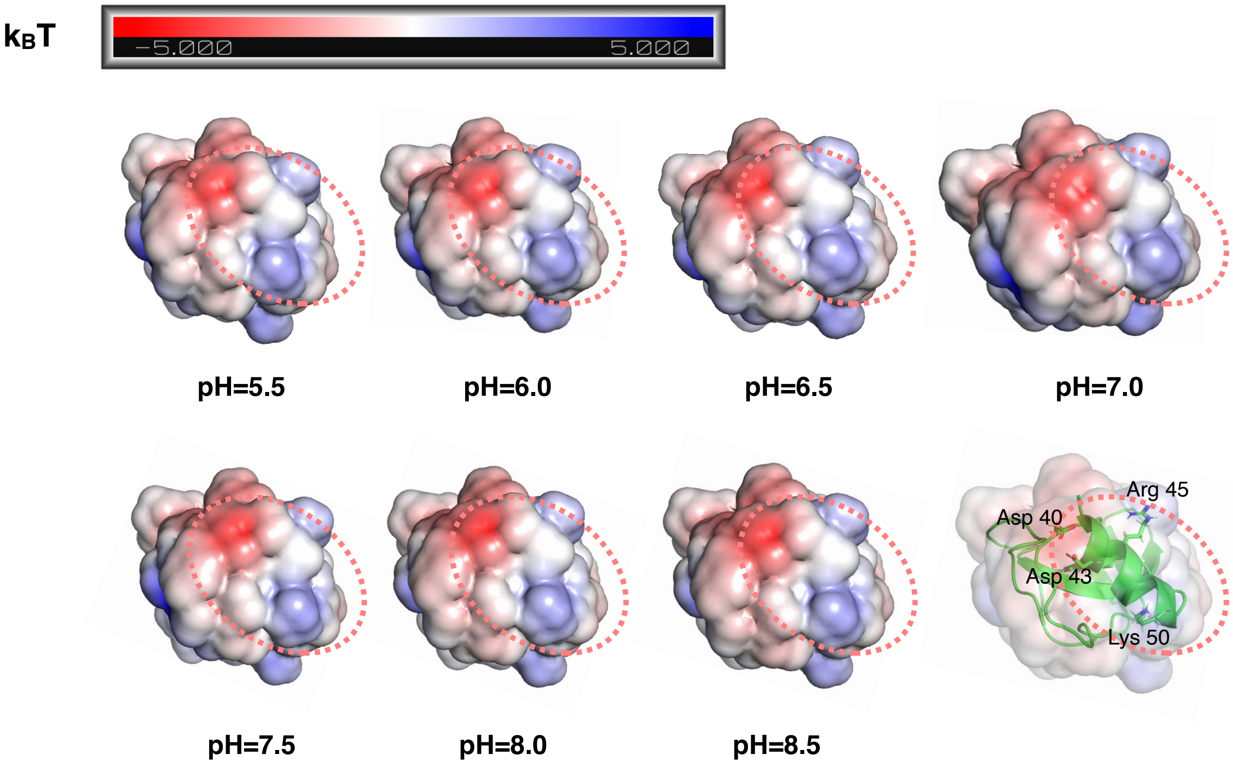

Supplementary Fig. 14 | The electrostatic potentials of HFBI at different pHs are mapped onto its solvent accessible surface (hydrophilic side). Red circles highlight the regions with solvent accessible residues. The electrostatic potentials are coloured from red (negative charge) to blue (positive charge) with the range of -5.0 ~ 5.0 k_BT.

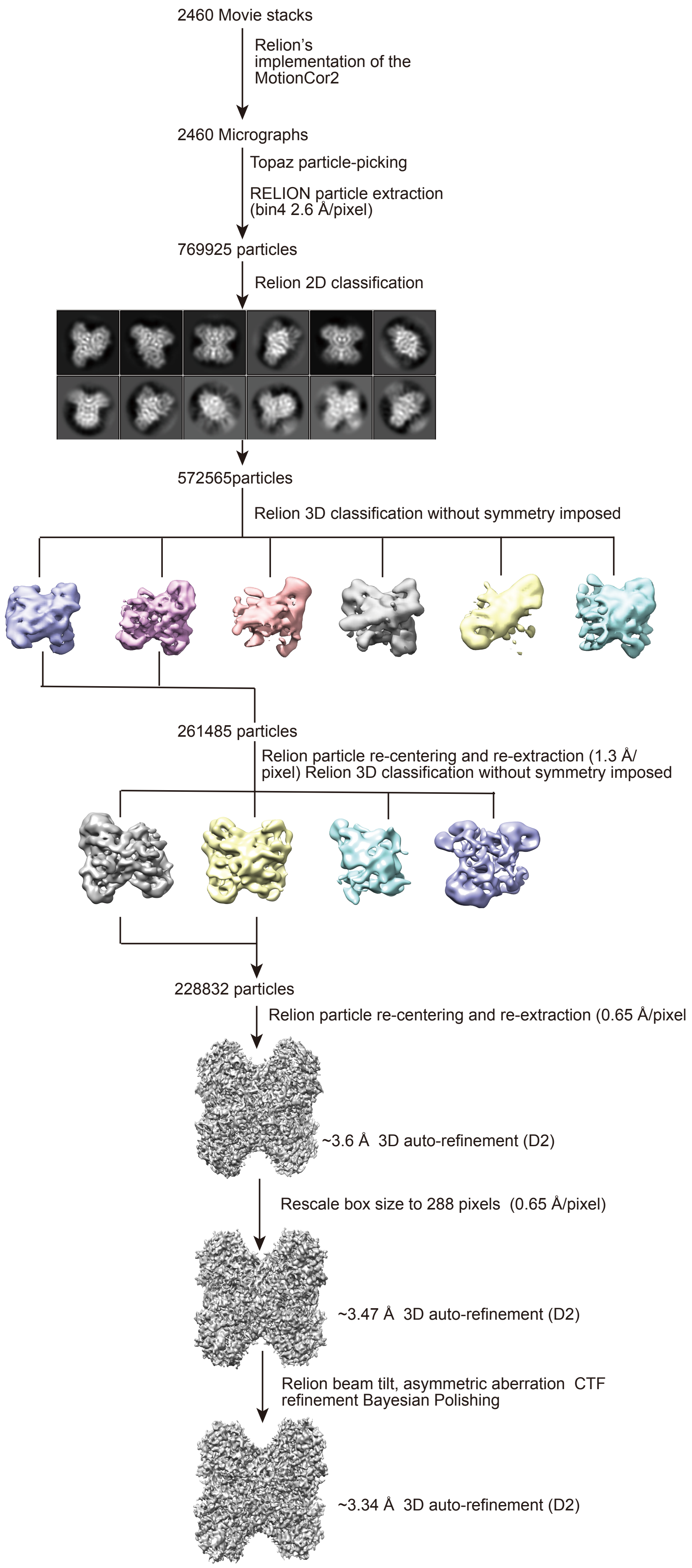

Supplementary Fig. 15 I Cryo-EM image processing workflow of aldolase cryo-vitrified using HFBI film covered ANTA foil grid.

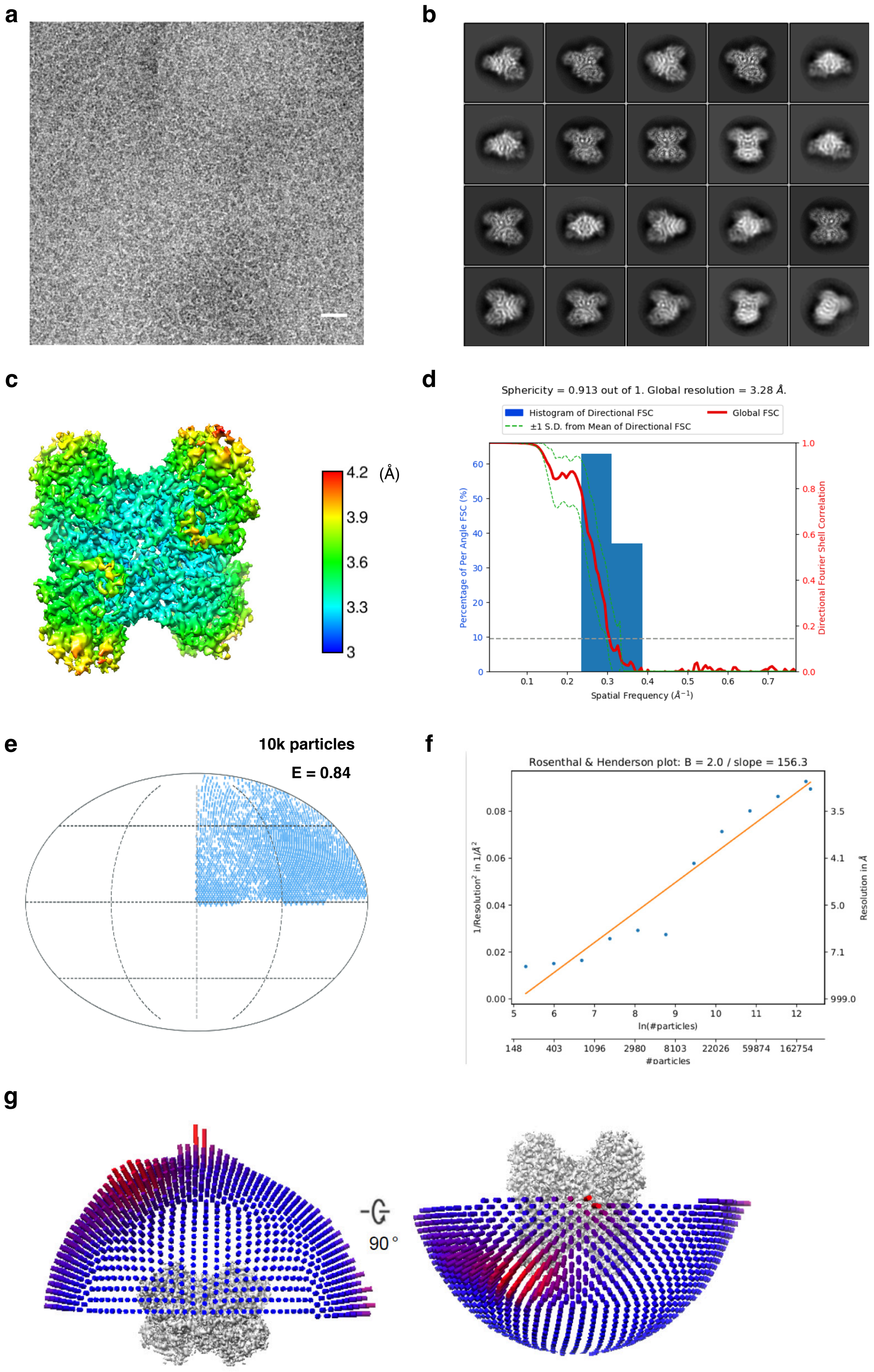
a
b
c
4.2 (Å)
3.9
3.6
3.3
3
d
Sphericity = 0.913 out of 1. Global resolution = 3.28 Å.
Histogram of Directional FSC
±1 S.D. from Mean of Directional FSC
Global FSC
Percentage of Per Angle FSC (%)
Directional Fourier Shell Correlation
Spatial Frequency (Å⁻¹)
e
10k particles
E = 0.84
f
Rosenthal & Henderson plot: B = 2.0 / slope = 156.3
1/Resolution² in 1/Å²
Resolution in Å
ln(#particles)
#particles
g
90°

Supplementary Fig. 16 | Cryo-EM structure determination of aldolase cryo-vitrified using HFBI film covered ANTA foil grid. **a**, The representative cryo-EM micrograph of aldolase. Scale bar, 20 nm. **b**, The representative 2D class averages. **c**, Cryo-EM map of aldolase is coloured according to the local resolution (from 4.2 Å in red to 3.0 Å in blue). **d**, 3D-FSC plot of the cryo-EM reconstruction. **e**, The orientation distribution of aldolase particles with the cryo-EF value of 0.84 calculated using 10,000 particles. **f**, Rosenthal-Henderson plot with the estimated B-factor of 156.3 Å. **g**, Plots showing the Euler angle distribution of the particles.

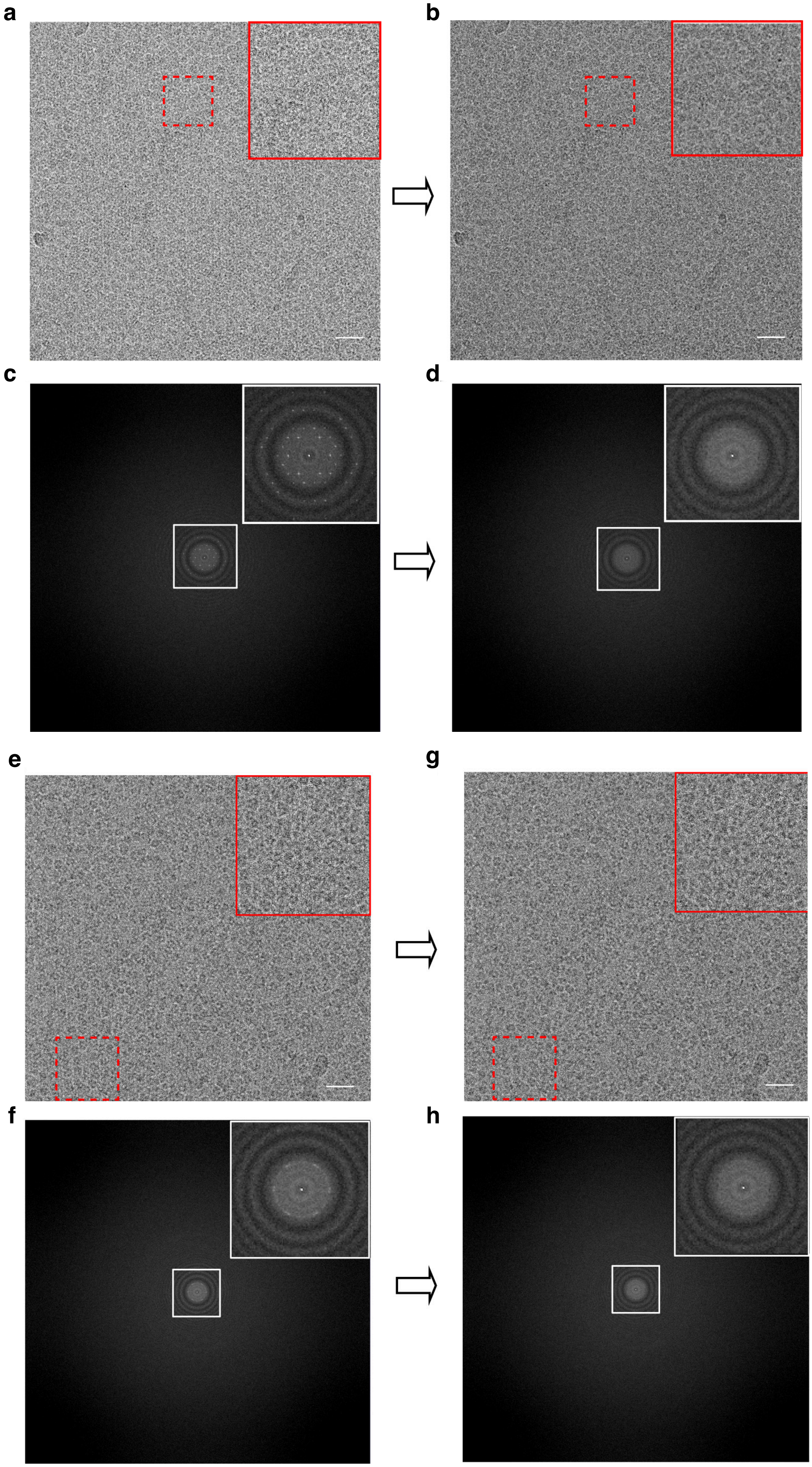
a
b
c
d
e
g
f
h

Supplementary Fig. 17 | Subtracting HFBI film background by Fourier lattice filtering. a-d, The subtraction example with the monocrystal of HFBI film. **e**-**h**, The subtraction example with the polycrystal of HFBI film. In both cases, the representative micrographs of haemoglobin (**a** and **e,** Scale bar, 20 nm) are shown with the selected regions (dashed red squares) zoomed in at the insets (red squares). Their corresponding power spectrums are displayed below (**c** and **f**) with the central regions zoomed in at the insets showing the diffraction spots of HFBI film background. The diffraction spots were removed by using Fourier filtering algorithm (for details see Methods) and the resulting power spectrums are displayed aside (**d** and **h**) with the corresponding central regions zoomed in at the insets showing the removal of the diffraction spots. After diffraction spots removing, inverse Fourier transformations were performed to get the final micrographs (**b** and **g**). The selected regions (dashed red squares) are zoomed in (red squares) to show the removal of the HFBI film background.

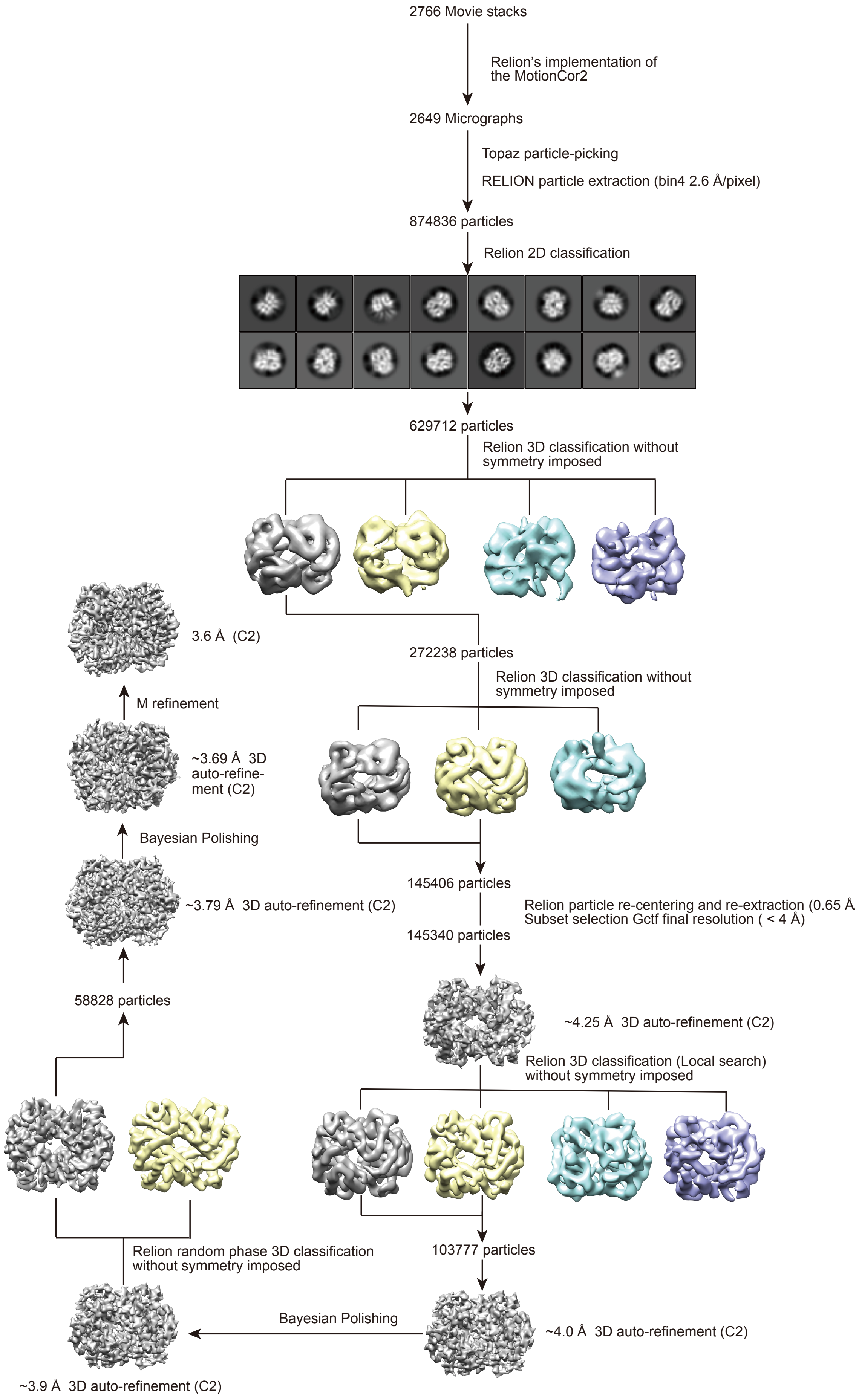

Supplementary Fig. 18 | Cryo-EM image processing workflow of haemoglobin cryo-vitrified using HFBI film covered ANTA foil grid.

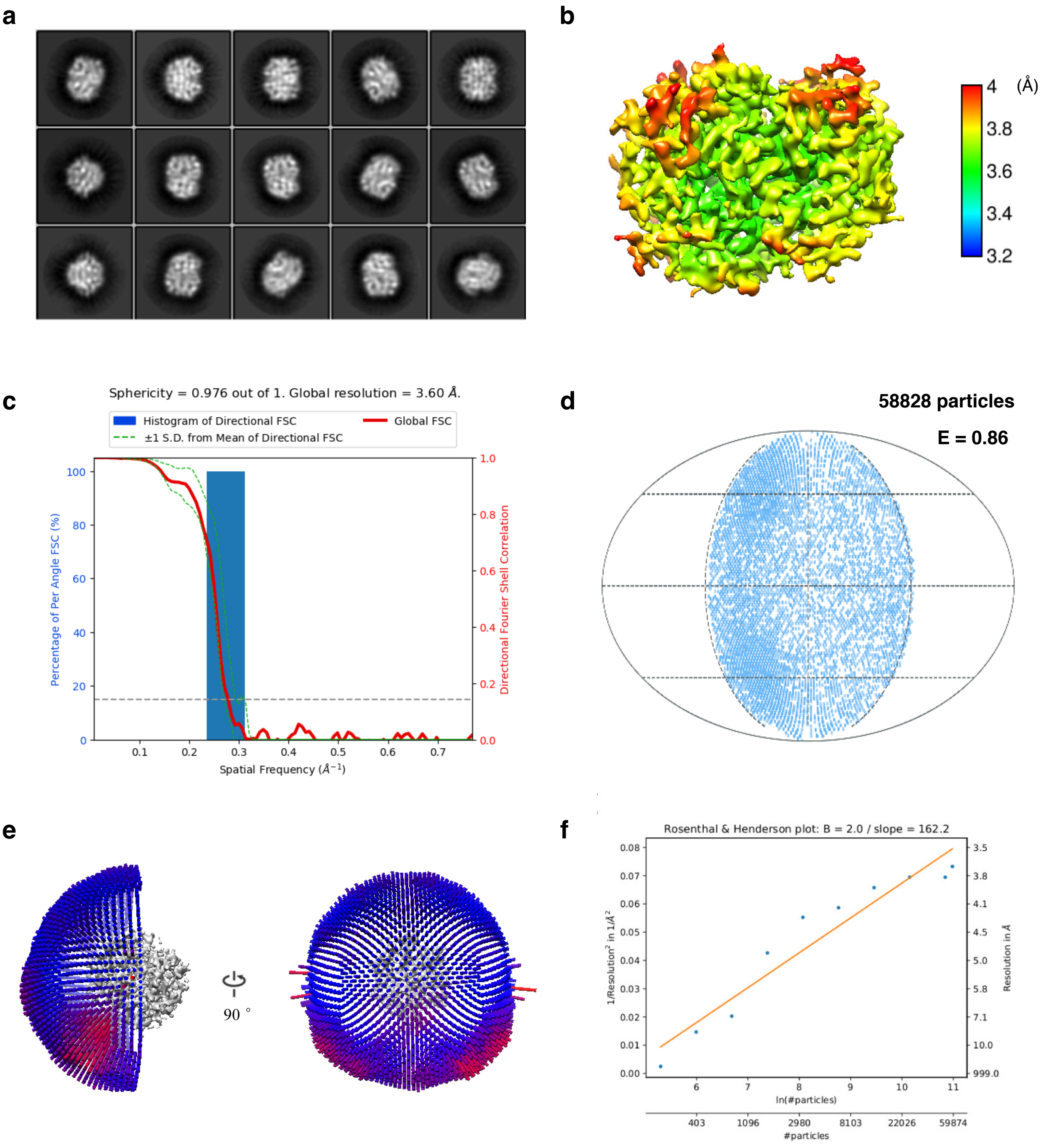

Supplementary Fig. 19 I Cryo-EM structure determination of haemoglobin cryo-vitrified using HFBI film covered ANTA foil grid. a, The representative 2D class averages. **b**, Cryo-EM map of haemoglobin is coloured according to the local resolution (from 4.0 Å in red to 3.2 Å in blue). **c**, 3D-FSC plot of the cryo-EM reconstruction. **d**, The orientation distribution of haemoglobin particles with the cryo-EF value of 0.86 calculated using 58,828 particles. **e**, Plots showing the Euler angle distribution of the particles. **f**, Rosenthal-Henderson plot with the estimated B-factor of 162.2 Å.

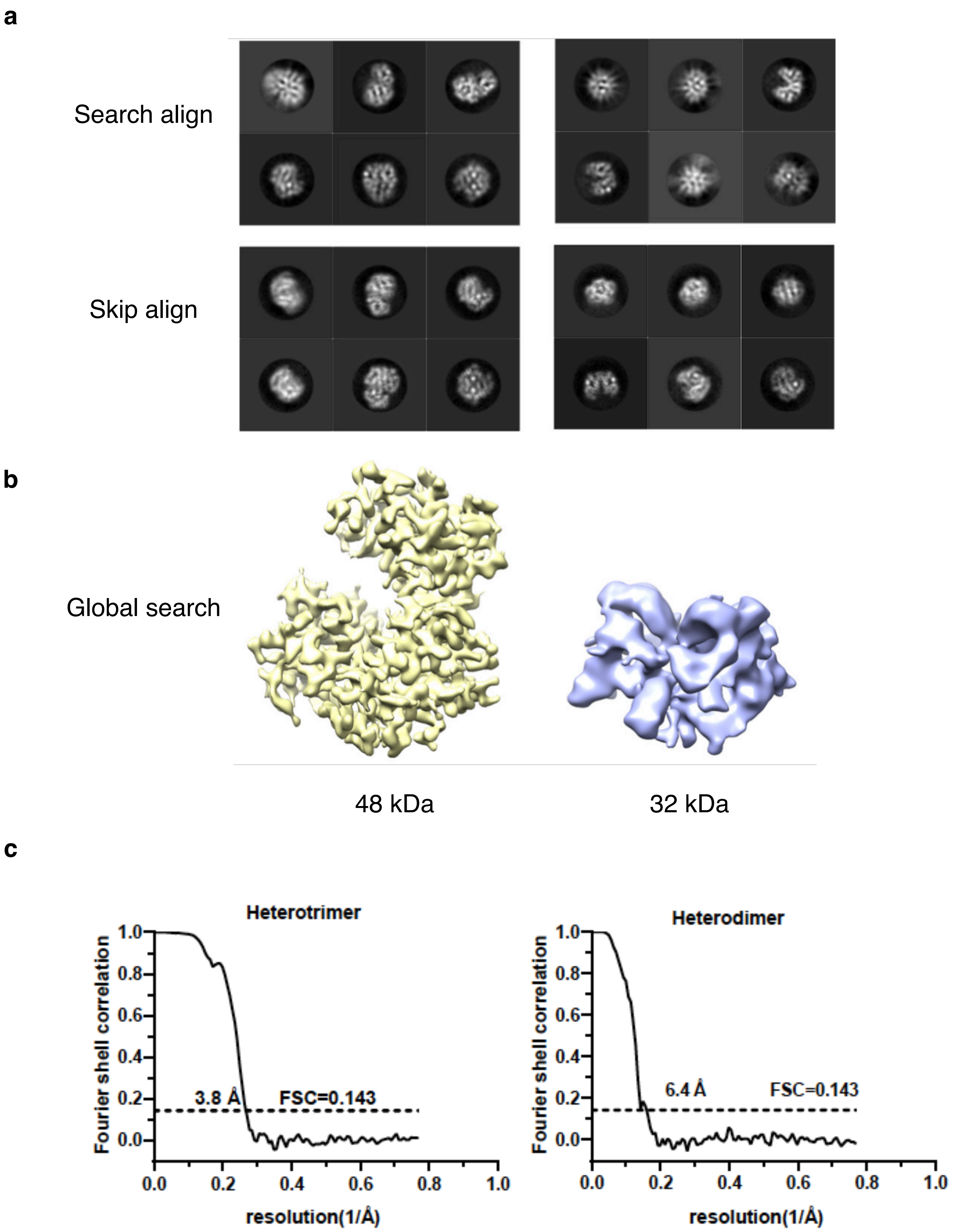

Supplementary Fig. 20 l Reconstructions of *in silico* subtracted haemoglobin sub-particles. a, 2D class averages of sub-particles (Left, the hetero trimer; Right, the hetero dimer) with the global parameter search (search align) or with the pre-determined angular information (skip align). **b**, 3D reconstructions of sub-particles with the global parameter search. The map of hetero trimer (48 kDa) is coloured in yellow, and the map of hetero dimer (32 kDa) is in wathet blue. **c**, Gold standard FSC curves of the reconstructions of hetero trimer (left) and hetero dimer (right), respectively.

Supplementary Table 1. Statistics of cryo-EM data collection and processing, and model building, refinement and validation

**Protein**	Apoferritin	Catalase	GDH	HA trimer	Aldolase	Haemoglobin
**Data collection and processing**						
Type of electron microscope	Talos Arctica	Titan Krios	Titan Krios	Talos Arctica	Titan Krios	Talos Arctica
Type of direct detection camera	Gatan K2 camera	Gatan K2 camera	Gatan K2 camera	Gatan K2 camera	Gatan K2 camera	Gatan K2 camera
Voltage (kV)	200	300	300	200	300	200
Nominal magnification	165,000	165,000/215,000	215,000	165,000	215,000	215,000
Electron exposure ($e^-/Å^2$)	50	60	60	60	70	70
Defocus range (μm)	–0.6 ~ –2.0	–0.6 ~ –1.6	–0.6~ –1.6	–0.8~ –2.0	–0.6~ –1.6	–0.6~ –1.6
Pixel size (Å)	0.588	0.82/0.65[a]	0.65	0.76	0.65	0.65
Symmetry imposed	O	D2	D3	C3	D2	C2
Initial particle images (no.)	351,565	653,922[b]	35,587	1,213,983	769,925	874,836
Final particle images (no.)	108,183	195,077	18,419	196,464	228,832	58,828
Map resolution (Å)	1.96	2.29	2.26	2.56	3.28	3.6
FSC threshold	0.143	0.143	0.143	0.143	0.143	0.143
Map resolution range (Å)	1.9 - 2.2	2.2 - 2.7	2.0 - 3.5	2.3-3.5	3.1 – 4.0	3.5 – 4.0
Rosenthal-Henderson *B* factor ($Å^2$)	87.8	79.3	76	185.6	156.3	162.2
**Model building and refinement**						

Initial model (PDB code)	7K3V	1DGH	3JCZ	6WXB	6V20	5NI1
Model-to-map resolution (Å)	1.9	2.5	2.4	2.8	3.5	3.7
FSC threshold	0.5	0.5	0.5	0.5	0.5	0.5
Model composition						
Non-hydrogen atoms	38,201	16,430	23,292	22,884	10,476	4555
Protein residues	4128	1991	2976	1455	1372	574
Ligands	329	8	0	18	0	4
B factors (Å^2)						
Protein	4.46	24.46	29.7	47.99	57.56	36.75
Ligands	21.3	15.28	/	46.78	/	11.06
Water	3.43	/	/	/	/	/
R.m.s. deviations						
Bond lengths (Å)	0.002	0.002	0.002	0.003	0.002	0.003
Bond angles (°)	0.559	0.549	0.537	0.5	0.523	0.632
Validation						
MolProbity score	1.1	1.96	1.81	1.65	1.68	1.13
Clashscore	2.67	7.68	7.91	6.91	9.54	3.42
Rotamer outliers (%)	0	2.33	2.16	0	0	0
Ramachandran plot (%)						
Favored	97.79	96.17	97.37	96.05	96.99	98.23
Allowed	2.21	3.83	2.63	3.95	3.01	1.77
Disallowed	0	0	0	0	0	0

[a]For catalase, different datasets were collected with different magnification of 165,000 and 215,000, corresponding to different pixel size of 0.82 Å and 0.65 Å, respectively.
[b]Particles picked from different datasets with different pixel sizes. The particles were then combined using RELION 3.1.

Supplementary References

1. Gipson, B., Zeng, X., Zhang, Z.Y. & Stahlberg, H. 2dx--user-friendly image processing for 2D crystals. *J Struct Biol* **157**, 64-72 (2007).
2. Henderson, R., Baldwin, J.M., Downing, K.H., Lepault, J. & Zemlin, F. Structure of purple membrane from halobacterium halobium: recording, measurement and evaluation of electron micrographs at 3.5 Å resolution. *Ultramicroscopy* **19**, 147-178 (1986).
3. Zhang, K. Gctf: Real-time CTF determination and correction. *J Struct Biol* **193**, 1-12 (2016).
4. Yan, R. et al. Simultaneous determination of sample thickness, tilt, and electron mean free path using tomographic tilt images based on Beer-Lambert law. *Journal of structural biology* **192**, 287-296 (2015).
5. Kremer, J.R., Mastronarde, D.N. & McIntosh, J.R. Computer visualization of three-dimensional image data using IMOD. *Journal of structural biology* **116**, 71-76 (1996).
6. Terwilliger, T.C., Ludtke, S.J., Read, R.J., Adams, P.D. & Afonine, P.V. Improvement of cryo-EM maps by density modification. *Nat Methods* **17**, 923-927 (2020).
7. Adams, P.D. et al. PHENIX: a comprehensive Python-based system for macromolecular structure solution. *Acta Crystallogr D Biol Crystallogr* **66**, 213-221 (2010).
8. Ramirez-Aportela, E., Mota, J., Conesa, P., Carazo, J.M. & Sorzano, C.O.S. DeepRes: a new deep-learning- and aspect-based local resolution method for electron-microscopy maps. *IUCrJ* **6**, 1054-1063 (2019).
9. Wu, M., Lander, G.C. & Herzik, M.A., Jr. Sub-2 Angstrom resolution structure determination using single-particle cryo-EM at 200keV. *J Struct Biol X* **4**, 100020 (2020).
10. Tan, Y.Z. et al. Addressing preferred specimen orientation in single-particle cryo-EM through tilting. *Nat Methods* **14**, 793-796 (2017).
11. Zivanov, J. et al. New tools for automated high-resolution cryo-EM structure determination in RELION-3. *Elife* **7** (2018).
12. Noble, A.J. et al. Reducing effects of particle adsorption to the air-water interface in cryo-EM. *Nat Methods* **15**, 793-795 (2018).
13. Tan, Y.Z. & Rubinstein, J.L. Through-grid wicking enables high-speed cryoEM specimen preparation. *Acta Crystallogr D Struct Biol* **76**, 1092-1103 (2020).
