# Supplementary material for: A cryo-electron microscopy support film formed by 2D crystals of hydrophobin HFBI
Source: Nat Commun. 2021 Dec 14;12:7257. doi: 10.1038/s41467-021-27596-8 (PMC8671466; doi:10.1038/s41467-021-27596-8)
Supplement: Supplementary file 3 — Reporting Summary [file 41467_2021_27596_MOESM3_ESM.pdf]

nature research

Corresponding author(s): Fei Sun, Mingqiang Qiao.

Last updated by author(s): Nov 21, 2021

Reporting Summary

Nature Research wishes to improve the reproducibility of the work that we publish. This form provides structure for consistency and transparency in reporting. For further information on Nature Research policies, see our Editorial Policies and the Editorial Policy Checklist.

Statistics

For all statistical analyses, confirm that the following items are present in the figure legend, table legend, main text, or Methods section.

n/a	Confirmed	
☐	☒	The exact sample size (n) for each experimental group/condition, given as a discrete number and unit of measurement
☒	☐	A statement on whether measurements were taken from distinct samples or whether the same sample was measured repeatedly
☒	☐	The statistical test(s) used AND whether they are one- or two-sided<br>*Only common tests should be described solely by name; describe more complex techniques in the Methods section.*
☒	☐	A description of all covariates tested
☒	☐	A description of any assumptions or corrections, such as tests of normality and adjustment for multiple comparisons
☒	☐	A full description of the statistical parameters including central tendency (e.g. means) or other basic estimates (e.g. regression coefficient) AND variation (e.g. standard deviation) or associated estimates of uncertainty (e.g. confidence intervals)
☒	☐	For null hypothesis testing, the test statistic (e.g. F, t, r) with confidence intervals, effect sizes, degrees of freedom and P value noted<br>*Give P values as exact values whenever suitable.*
☒	☐	For Bayesian analysis, information on the choice of priors and Markov chain Monte Carlo settings
☒	☐	For hierarchical and complex designs, identification of the appropriate level for tests and full reporting of outcomes
☒	☐	Estimates of effect sizes (e.g. Cohen's d, Pearson's r), indicating how they were calculated

Our web collection on statistics for biologists contains articles on many of the points above.

Software and code

Policy information about availability of computer code

Data collection	Cryo-EM data were automatically collected using SerialEM3.7 at Center for Biological Imaging (CBI, http://cbi.ibp.ac.cn), Institute of Biophysics, Chinese Academy of Sciences.
Data analysis	Cryo-EM data were analyzed using RELION3.1, Gctf1.06, Gautomatch0.56, Topaz0.2.2, MotionCorr2, cryoSPARC3.0, EMAN2.3, COOT0.8.9.2, PHENIX (v1.19-4092), Pyem (v.0.5), UCSF-Chimera64-1.15, IMOD 4.9, ICON, AutoGDeterm.

For manuscripts utilizing custom algorithms or software that are central to the research but not yet described in published literature, software must be made available to editors and reviewers. We strongly encourage code deposition in a community repository (e.g. GitHub). See the Nature Research guidelines for submitting code & software for further information.

Data

Policy information about availability of data

All manuscripts must include a data availability statement. This statement should provide the following information, where applicable:

- Accession codes, unique identifiers, or web links for publicly available datasets
- A list of figures that have associated raw data
- A description of any restrictions on data availability

The atomic coordinates and cryo-EM density maps of apoferritin, catalase, glutamate dehydrogenase, HA trimer, aldolase and haemoglobin have been deposited in the RCSB Protein Data Bank (PDB) with the accession codes 7VD8, 7VD9, 7VDA, 7VDF, 7VDC and 7VDE and in the Electron Microscopy Data Bank (EMDB) with the accession codes EMD-31910, EMD-31911, EMD-31912, EMD-31916, EMD-31913 and EMD-31915, respectively.

Field-specific reporting

Please select the one below that is the best fit for your research. If you are not sure, read the appropriate sections before making your selection.

☒ Life sciences ☐ Behavioural & social sciences ☐ Ecological, evolutionary & environmental sciences

For a reference copy of the document with all sections, see nature.com/documents/nr-reporting-summary-flat.pdf

Life sciences study design

All studies must disclose on these points even when the disclosure is negative.

Sample size	Numbers of particles picked and processed and numbers of electron micrographs used for statistics have been described accordingly in the manuscript.
Data exclusions	Only wrongly picked and bad particles were excluded during CryoEM SPA image processing. There was no data exclusion in other experiments.
Replication	All the representative electron micrographs in this work were repeated to acquire at least three times independently, which all resulted similar results. SDS-PAGE and AFM experiments were repeated independently at least three times with similar results.
Randomization	Randomization has been taken care in the standard image processing package like RELION3.1, cryoSPARC3.0 etc.
Blinding	Not applicable in this work.

Reporting for specific materials, systems and methods

We require information from authors about some types of materials, experimental systems and methods used in many studies. Here, indicate whether each material, system or method listed is relevant to your study. If you are not sure if a list item applies to your research, read the appropriate section before selecting a response.

Materials & experimental systems

n/a	Involved in the study	
☒	☐	Antibodies
☒	☐	Eukaryotic cell lines
☒	☐	Palaeontology and archaeology
☒	☐	Animals and other organisms
☒	☐	Human research participants
☒	☐	Clinical data
☒	☐	Dual use research of concern

Methods

n/a	Involved in the study	
☒	☐	ChIP-seq
☒	☐	Flow cytometry
☒	☐	MRI-based neuroimaging
